# Modality differences in timing and the filled-duration illusion: Testing the pacemaker rate explanation

**DOI:** 10.3758/s13414-018-1630-8

**Published:** 2018-12-19

**Authors:** Emily A. Williams, Ezgi M. Yüksel, Andrew J. Stewart, Luke A. Jones

**Affiliations:** 1 Division of Neuroscience and Experimental Psychology, University of Manchester, Manchester, UK; 2 Department of Psychology, Middle East Technical University, Ankara, Turkey

**Keywords:** Interval timing, Sensory modalities, Filled-duration illusion, Scalar timing theory, Pacemaker, Individual differences

## Abstract

Performance in temporal difference threshold and estimation tasks is markedly less accurate for visual than for auditory intervals. In addition, thresholds and estimates are likewise less accurate for empty than for filled intervals. In scalar timing theory, these differences have been explained as alterations in pacemaker rate, which is faster for auditory and filled intervals than for visual and empty intervals. We tested this explanation according to three research aims. First, we replicated the threshold and estimation tasks of Jones, Poliakoff, and Wells (*Quarterly Journal of Experimental Psychology, 62,* 2171–2186, 2009) and found the well-documented greater precision for auditory than visual intervals, and for filled than for empty intervals. Second, we considered *inter*-individual differences in these classic effects and found that up to 27% of participants exhibited opposite patterns. Finally, we examined *intra*-individual differences to investigate (i) whether thresholds and estimates correlate within each stimulus condition and (ii) whether the stimulus condition in which a participants’ pacemaker rate was highest was the same in both tasks. Here we found that if pacemaker rate is indeed a driving factor for thresholds and estimates, its effect may be greater for empty intervals, where the two tasks correlate, than for filled intervals, where they do not. In addition, it was more common for participants to perform best in different modalities in each task, though this was not true for ordinal intra-individual differences in the filled-duration illusion. Overall, this research presents several findings inconsistent with the pacemaker rate explanation.

**Electronic supplementary material:**

The online version of this article (10.3758/s13414-018-1630-8) contains supplementary material, which is available to authorized users.

The domain of interval timing by humans has historically been under-researched as compared to other perceptual domains. A key reason for this is that, although humans possess very sensitive discrimination for duration (with difference thresholds as low as 10 ms; Morrongiello & Trehub, 1987), there is no sensory organ for time. This forces explanations (or models) of timing to draw on hidden processes more heavily than is necessary for other sensory systems, such as vision and hearing.

To date, one of the most successful of these models has focused on the idea that humans possess an internal clock of a pacemaker–accumulator type, such as in scalar timing theory (Gibbon, 1977; Gibbon & Church, 1984; Gibbon, Church, & Meck, 1984). The *pacemaker* is said to emit pulses at a given rate, which are sent to the *accumulator* when an attentional *switch* is closed. The accumulated contents are said to increase linearly with the interval being timed, forming the basis of its perceived duration. Further memory and decision process are usually implicated, depending on the demands of the task (for a detailed exposition of these assumptions, see Gibbon & Church, 1984).^1^ In scalar timing theory, the pacemaker is said to follow a Poisson process and to drift from trial to trial, in order to obey the scalar property (i.e., increased variability of timing judgments at longer time intervals).

Despite the apparent accuracy of the internal clock, many nontemporal characteristics of stimuli have been found to affect judgments of duration (see Matthews & Meck, 2016, for a recent review). For example, classic work by Goldstone and colleagues showed that intervals were judged to be longer when presented as a sound than when presented as a light (Goldstone, Boardman, & Lhamon, 1959; Goldstone & Goldfarb, 1964). This effect has more recently been found using the verbal estimation task,^2^ in which participants’ typed estimates are greater for auditory than for visual intervals (Jones, Poliakoff, & Wells, 2009; Wearden, Edwards, Fakhri, & Percival, 1998; Wearden, Todd, & Jones, 2006). Proponents of scalar timing theory suggest that this effect is due to the pacemaker pulsing at a slower rate for visual than for auditory intervals, resulting in fewer accumulated pulses over the same period (Jones et al., 2009; Wearden et al., 1998; Wearden et al., 2006).

A key signature of a putative change in pacemaker rate in verbal estimation is the “slope effect.” When stimulus durations are plotted against estimates of those durations, the difference between two conditions (i.e., auditory and visual) manifests as a difference in slope (see Fig. 1). When the rate of the pacemaker decreases, fewer pulses are emitted per second, leading to a greater difference between the experimental and control conditions with increasing stimulus duration. This is in contrast to an “intercept effect,” which arises due to the latency of the switch to start and stop timing. In this way, intercept effects are additive—that is, independent of stimulus duration (see the dotted line in Fig. 1)—but can occur in combination with slope effects (the dot-dashed line in Fig. 1).

**Fig. 1:**
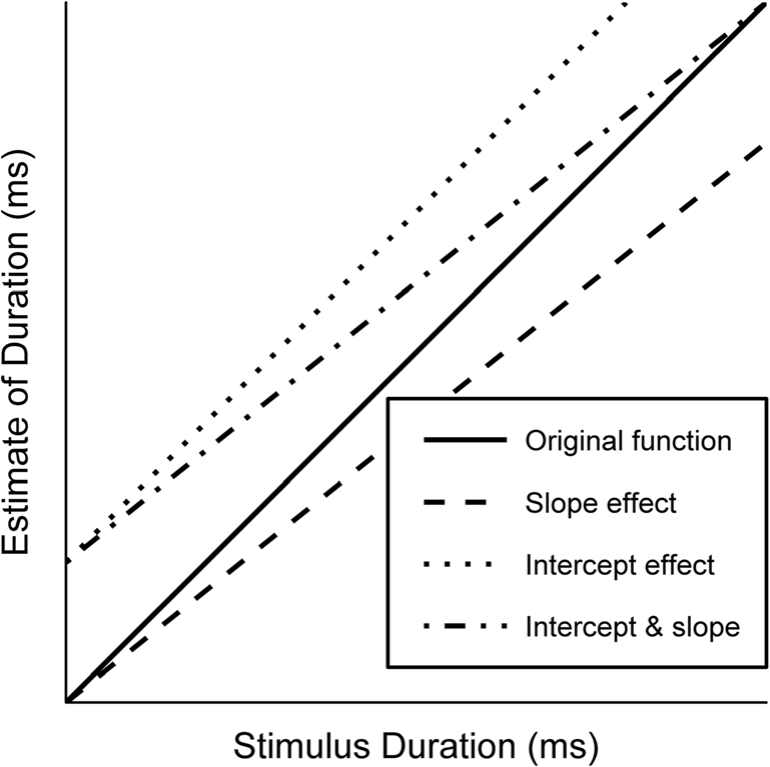
Examples of a slope effect (dashed line), an intercept effect (dotted line) and a combination of the two (dash-dotted line), all relative to the original function (solid line). Note that slopes can also increase, and intercepts can decrease

Matthews (2011) sought to investigate whether slope effects are a necessary artifact of verbal estimation, due to their apparent ubiquity. He found that it was possible to obtain a pure intercept effect in the absence of a slope effect in this paradigm. However, Matthews also found that slope effects could manifest when a change in pacemaker rate would be inappropriate—that is, between two empty intervals with the same onset marker, but different offset markers. For this reason, Matthews urged caution when using verbal estimation to investigate the pacemaker, since the task can erroneously produce slope effects. Wearden, Williams, and Jones (2017) noted that the inappropriate slope effect found by Matthews differed from the slope effects found in previous research, in which the differences in estimates had increased as a function of increasing stimulus duration. In Matthews’s work, the differences in estimates decreased as stimulus duration increased. Therefore, this finding does not necessarily contradict the application of the pacemaker explanation to previous work, but it does indeed highlight the need for further study into the mechanisms that produce slope effects in verbal estimation.

Other tasks that have demonstrated auditory–visual differences include temporal generalization (Jones et al., 2009; Klapproth, 2002; Wearden et al., 1998), in which participants judge whether presented durations are the same as a previously presented standard duration, and temporal bisection (Droit-Volet, Tourret, & Wearden, 2004; Penney, Gibbon, & Meck, 2000; Wearden et al., 2006), in which participants judge whether durations are more similar to a “short” or a “long” standard duration. However, the most popular task used to investigate auditory–visual differences in timing is the temporal difference threshold task^3^ (also “duration discrimination”; Buffardi, 1971; Grondin, 1993; Grondin, Meilleur-Wells, Oulette, & Macar, 1998; Jones et al., 2009; Marks, 1987; Ulrich, Nitschke, & Rammsayer, 2006).

Although the temporal difference threshold task has not been modeled according to scalar timing theory (unlike verbal estimation [Wearden, 2015], temporal generalization [Droit-Volet, Clément, & Wearden, 2001], or temporal bisection [Wearden, 1991]), the pacemaker explanation has also been applied to this task. It has been suggested that thresholds are significantly lower for auditory than for visual intervals because the pacemaker runs faster for auditory than for visual intervals (Jones et al., 2009). Indeed, it is often argued that a faster pacemaker would lead to higher temporal sensitivity (Rammsayer, 2008; Rammsayer & Grondin, 2000; Troche & Rammsayer, 2011). However, it is not clear what effect on thresholds scalar timing theory would specifically predict, due to the trial-by-trial variability of the pacemaker. Nevertheless, it would be interesting to test this simple application of a pacemaker explanation to the temporal difference threshold task. The collective incidence of the auditory–visual difference across verbal estimation, temporal bisection, and temporal generalization is consistent with the idea of a difference in pacemaker rate. It therefore appears that the pacemaker rate is determined by the modality of the stimulus and that the effect is independent from the demands of different tasks.

The judgment of tactile intervals is often overlooked, as compared with auditory and visual intervals, probably due to the relative difficulty of controlling stimulus presentation. The research that has been conducted has generally found that judgments and sensitivity for tactile intervals fall somewhere between those for auditory and visual intervals (Buffardi, 1971; Goodfellow, 1934; Westheimer, 1999). In one study, Jones et al. (2009) presented auditory, tactile, and visual intervals during temporal difference threshold and verbal estimation tasks. Although the mean thresholds and estimation slopes for tactile intervals fell between those for auditory and visual intervals, performance for tactile and visual intervals did not differ significantly in either task. It is pertinent that this null effect was found in both tasks, though different samples of participants completed each task. However, having the same sample of participants complete both the threshold and estimation tasks would allow for the investigation of *intra*-individual differences between the tasks. If both tasks *are* strongly determined by pacemaker rate, we would expect (i) thresholds and estimation slopes to correlate within each modality, and (ii) the modality in which a participant performs “best” to probably be the same in both tasks. We will therefore investigate intra-individual differences in modality differences to test these assumptions.

Another well-researched temporal illusion concerns the difference in judgments of filled and empty durations, often termed the “filled-duration illusion.” The duration of a filled interval is marked by a continuous stimulus, whereas the duration of an empty interval is marked by the gap between two brief stimuli. Temporal difference thresholds have been found to be higher for empty than for filled intervals, indicating a lower sensitivity to duration (Rammsayer & Lima, 1991). The presence of this effect is largely dependent on the duration of the intervals and the psychophysical procedure, among other factors (see Rammsayer, 2010, for a review). In addition, estimates of empty intervals are approximately 40% shorter than those for filled intervals, regardless of stimulus duration (from 77 to 1,183 ms; Wearden, Norton, Martin, & Montford-Bebb, 2007). Wearden and colleagues interpreted this as a reduction in the pacemaker rate for empty as compared to filled intervals.

The importance of examining *inter*-individual differences within the filled-duration illusion was highlighted by Hasuo and colleagues (Hasuo, Nakajima, Tomimatsu, Grondin, & Ueda, 2014; Hasuo, Nakajima, & Ueda, 2011), who found distinct subgroups of participants who exhibited different sizes, and sometimes reversals, of the filled-duration illusion. Hasuo et al. (2014) found that the participants who showed a pronounced difference between filled and empty intervals using the method of adjustment were not necessarily the same participants who exhibited the illusion in verbal estimation. Exploring the illusion at an individual level could be used to test the pacemaker explanation. It will be of interest to investigate whether, if a participant were to show a reverse effect in thresholds, they would also show a reverse effect in estimation slopes.

The cross-task pattern of better performance for filled than for empty intervals and the application of the pacemaker explanation lead us to consider the same questions asked regarding modality differences—namely, whether thresholds and slopes are related within each stimulus type, and whether participants achieve their highest performance in a congruent (i.e., the same) stimulus type across tasks (ordinal intra-individual differences). Heeding the work of Hasuo and colleagues, we will also investigate the pervasiveness of modality differences and the filled-duration illusion in our sample. Indeed, a recent review by Matthews and Meck (2014) highlighted the need for the consideration of (inter-)individual differences, “even for ‘classic’ effects” (p. 435).

Similar cross-task work has been conducted by Rammsayer and Brandler (2004), who performed correlational and principal components analyses across a range of timing tasks. The authors found positive correlations between performance on temporal difference threshold, temporal generalization, and temporal order judgment tasks. The results of their principal components analyses suggested that these tasks were informed by “a common pacemaker-based interval timing mechanism” (p. 115). More recently, Rammsayer and Brandler (2007) investigated the relationship between these tasks and psychometric *g* (an index of general intelligence), finding significant correlations between each of the three tasks and participants’ psychometric *g*. Therefore, although investigating correlations between timing tasks is not new, the relationship between thresholds and estimation tasks has yet to be investigated. Though arguably the two tasks tap into different timing mechanisms, the pacemaker explanation has been applied to both. In addition, it could be argued that since thresholds have been found to correlate with several other timing tasks and a measure of intelligence, it would be reasonable to expect thresholds to correlate with temporal estimation slopes. It is therefore of interest to test the application of the pacemaker explanation to each of the tasks by replicating the procedure of Jones et al. (2009) in order to investigate inter- and intra-individual differences in performance for the auditory, tactile, and visual modalities as well as for filled and empty (auditory) intervals.

We posited three Research Aims:

1. To closely replicate Jones et al.’s (2009) Experiment 1 (temporal difference thresholds) and Experiment 2 (verbal estimation), investigating modality differences, and to repeat the procedure with filled and empty intervals. Our Experiment 1 investigated modality differences in temporal difference thresholds (Part A) and verbal estimation (Part B), and our Experiment 2 then repeated these tasks for filled and empty intervals.
2. To investigate inter-individual differences: How pervasive are the classic effects of stimulus modality (auditory, tactile, and visual) and stimulus type (filled vs. empty) on thresholds and estimation slopes? Inter-individual differences are rarely investigated within the field of time perception, but their exploration provides a more nuanced understanding of the main effects commonly reported (Hasuo et al., 2014; Matthews & Meck, 2014).
3. To investigate intra-individual differences: How do the two tasks relate to each other at the participant level?
  - The application of the pacemaker explanation to both tasks suggests that thresholds and estimation slopes might correlate, due to the common hypothesized mechanism. Is performance within each stimulus modality (or type) correlated between timing tasks?
  - If the pacemaker rate arising from a certain condition were consistent across tasks, this would suggest that the condition in which a participants’ pacemaker is fastest would be the same in both tasks. We therefore investigated whether the condition in which a participant performed best (e.g., auditory) was consistent across tasks, in addition to considering intermediate and worst performance (“ordinal analyses”).

## Experiment 1A: Modality differences in temporal difference thresholds

Since modality differences in both temporal difference threshold and verbal estimation tasks have been argued to be due to differences in pacemaker rate (Jones et al., 2009), one would expect some level of correlation between performance on these tasks. To investigate this, we began with a replication of the temporal difference threshold task from Jones and colleagues, who found lower temporal difference thresholds for auditory intervals, followed by tactile and then visual intervals (although there was no significant difference between the tactile and visual thresholds). We predicted similar results.

We also considered the pervasiveness of these modality differences in thresholds (Research Aim 2) by investigating the proportion of participants who have lower thresholds for auditory than for visual intervals. In addition, the frequencies of the six possible modality patterns (e.g., steepest for auditory, intermediate for tactile, and shallowest for visual intervals) were investigated. Following the results of Jones et al. (2009), we anticipated that the lowest thresholds would be more common for auditory than for tactile and visual intervals, and that the most common pattern overall would be the example given in the last sentence.

### Method

#### Participants

Fifty-two right-handed participants^4^ (staff and students of the University of Manchester and members of the general population) completed Experiments 1A and 1B in a random order and received £10 for their time. These participants (mean age 27 years, ranging from 20 to 56 years old) had normal or corrected-to-normal vision and normal hearing.

Participants also completed a temporal order judgment task, the results of which are beyond the remit of this article. The full testing session lasted approximately 1 h 30 min, and the temporal difference threshold, verbal estimation, and temporal order judgment tasks were completed in a random order.

#### Design

The independent variable was the modality of the stimulus (auditory, tactile, or visual). The dependent variable was participants’ resulting temporal difference thresholds, calculated as the mean difference between the standard and the comparison durations over the last 20 trials.

#### Apparatus and materials

Participants sat at a table in a dark room with their head resting on a chin rest. A PC presented the experiments, written in E-Prime (Psychology Software Tools, Pittsburgh, PA). A 17-in. flat-screen Samsung Syncmaster monitor stood at a distance of 60 cm from the chin rest. Participants’ eyes were level with the top of the monitor, and the fixation cue and questions were displayed 20° below eye level. A black foam grip (5.5 × 9.5 × 4.5 cm) was secured to the table 30 cm in front of participants, on the center line. Behind the grip was a Philips portable speaker, which presented the auditory stimuli (500-Hz sine-wave tones), and to the left of the grip was a numerical keypad (8.5 × 12 cm) for use with the left (nondominant) hand.

The grip housed an Oticon-A (100-Ω) bone conductor with a vibrating surface of 1.6 × 2.4 cm. The bone conductor was inset into the foam in the index finger position when gripped with the dominant right hand, and was driven by a 500-Hz sine-wave signal through a TactAmp 4.2 amplifier (Dancer Design). Visual stimuli were presented via a 6-mm green LED light (87 cd/m^2^), embedded in a black plastic casing (4 × 4 × 1.75 cm) and attached centrally on top of the foam grip. The LED was 16° below the fixation cue (36° below eye level) and 32 cm in front of participants. Participants wore 3M Peltor ear protectors (SNR^5^ = 37 dB) with inset earphones, which played white noise (56 dB) during each block in order to mask the sound of the vibrations. All participants agreed that the auditory stimuli from the speaker (presented at a constant volume for all participants) were clearly audible through the background of white noise and the ear protectors.

#### Procedure

Participants completed a 50-trial adaptive threshold task in each of the three modalities in a random order. Each trial began with the presentation of a fixation cross for 500–1,000 ms. The standard duration was 700 ms, whereas the comparison duration began at 1,000 ms.^6^ After each trial, participants were asked to press “1” if they judged the first interval to be longer, or “2” if they judged the second interval to be longer. The order of the standard and comparison durations was counterbalanced between trials, and participants were not told that the duration of one of the intervals was consistent across trials. A 500- to 1,000-ms delay occurred between the two intervals, and a 125- to 250-ms delay followed the second stimulus.

Threshold procedures were controlled by the weighted up–down staircase method (Kaernbach, 1991), which allows the upward steps (*S*_up_) and downward steps (*S*_down_) to be of different sizes. Kaernbach stated the equilibrium point for *Xp* as *S*_up_*p* = *S*_down_ (1–*p*). In the present task, convergence to X75 was desired, which relates to the ability to correctly distinguish between durations (with a difference of *X*) 75% of the time. This necessitated a “3-down, 1-up” staircase, in which the difference between the tones was decreased by one step size if participants were correct, but was increased by three step sizes if participants were incorrect. The step size was initially set to 15 ms, but it decreased to 10 ms after 30 trials in order to find the threshold more precisely. Five practice trials were presented at the start of each task, but advancement was not contingent on performance. This experiment took approximately 18 min to complete.

### Results^7^

#### Temporal difference thresholds

**Fig. 2:**
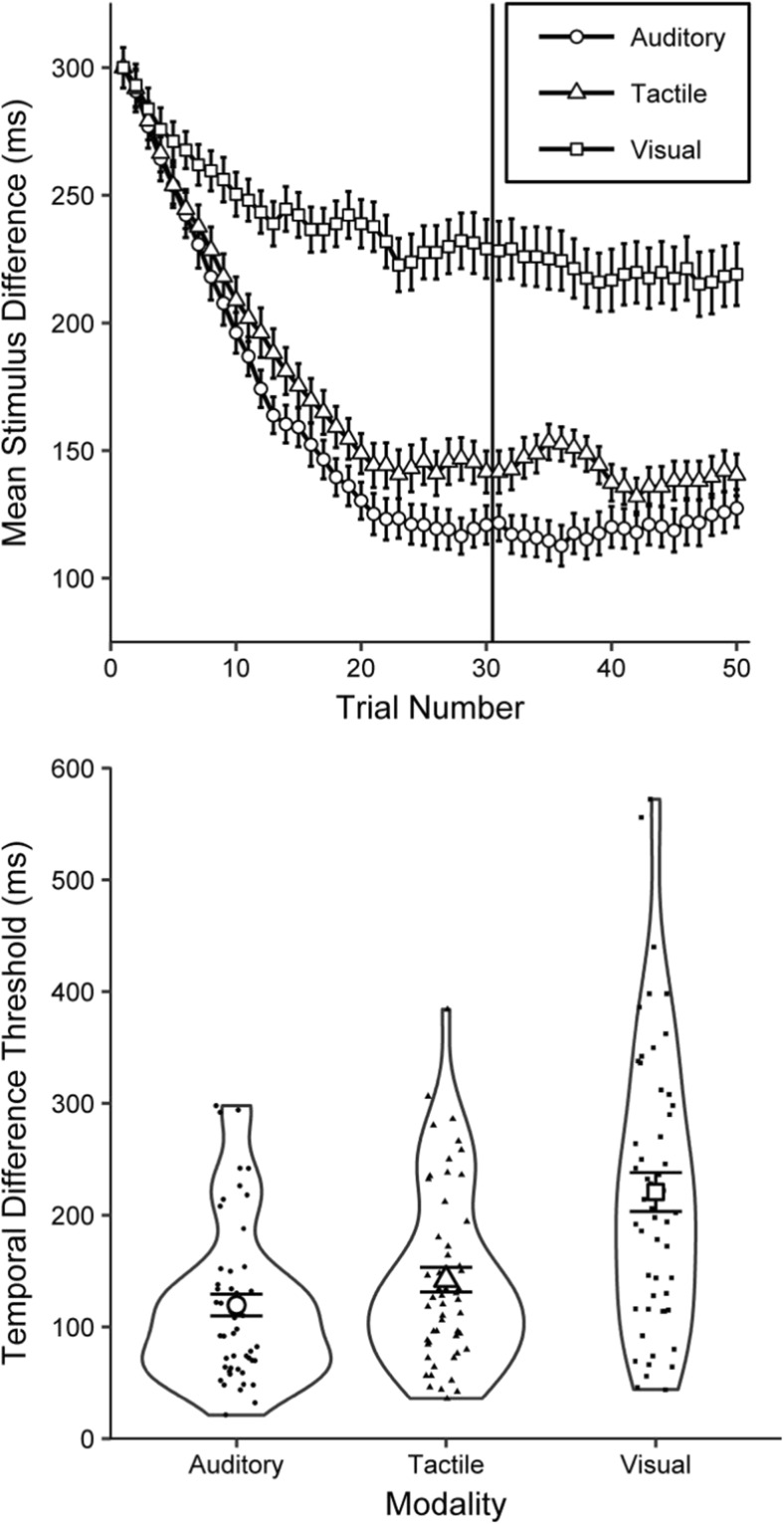
Threshold performance for Experiment 1A. (Upper panel) Mean difference between the standard and comparator across the 50 trials for each modality. The vertical solid line separates the last 20 trials, over which the step size was reduced and the temporal difference thresholds were calculated. Error bars denote within-participant standard errors (Morey, 2008). (Lower panel) Raw data, descriptive statistics, and inferential test (RDI) plots presenting the mean temporal difference thresholds for auditory, tactile, and visual intervals. The mean for each condition is presented as the larger empty shape, with error bars indicating standard errors. Smaller filled shapes denote the threshold values for each participant in each modality. The distribution for each modality is mirrored to form an outline around each mean

Outliers were defined as thresholds greater than 600 ms (twice the starting difference^8^), which would suggest an inability to perform the task. However, no participants had thresholds above this value, giving a full sample of 52 participants. The upper panel of Fig. 2 shows the mean differences between the standard and comparator durations across the 50 trials for the three modalities.

Inspection of the upper panel of Fig. 2 suggests that participants found the task easiest for auditory intervals, followed closely by tactile intervals, whereas distinguishing between durations of visual intervals appears to have been more difficult. The lower panel of Fig. 2 presents the resulting temporal difference thresholds. Inspection suggests higher thresholds for visual intervals and lower thresholds for auditory and tactile intervals, with marginally lower thresholds for auditory than tactile intervals. The mean visual threshold was 85% higher than the mean auditory threshold. Table 1 compares the mean thresholds and standard deviations for each modality to those found by Jones et al. (2009). A one-way repeated measures analysis of variance (ANOVA) showed a significant difference between the thresholds for the different modalities, *F*(1.64, 83.41) = 30.89, *p* < .001, *η*_p_^2^ = .377.^9^ Post hoc analyses (Holm–Bonferroni corrected^10^) and concurrent Bayesian *t* tests^11^ with default prior scales (Jeffreys, 1961; Wagenmakers et al., 2018) confirmed that the thresholds for visual intervals were significantly higher than those for auditory (*a* = .017), *t*(51) = 6.51, *p* < .001, *BF*_+0_ = 816,972, *d* = 0.90, and tactile (*a* = .025), *t*(51) = 5.45, *p* < .001, *BF*_+0_ = 22,819, *d* = 0.76, intervals. In addition, the thresholds for auditory intervals were significantly lower than those for tactile intervals (*a* = .050), *t*(51) = 2.29, *p* = .026, *BF*_–0_ = 3.21, *d* = 0.32.

**Table 1:** Comparison of the means and standard deviations of our thresholds (in milliseconds) and the accompanying coefficients of variation to those of Jones et al. (2009)

Threshold	Jones et al. (2009)			The present work		
	*M*	*SD*	CV	*M*	*SD*	CV
Auditory	103.25	56.73	0.55	119.46	70.29	0.59
Tactile	160.38	66.34	0.41	142.20	79.74	0.57
Visual	196.76	88.61	0.45	220.69	125.43	0.56

*M* = mean, *SD* = standard deviation, CV = coefficient of variation (*SD*/*M*)

#### Research Aim 2: Exploration of interindividual differences in auditory, tactile, and visual thresholds

**Fig. 3:**
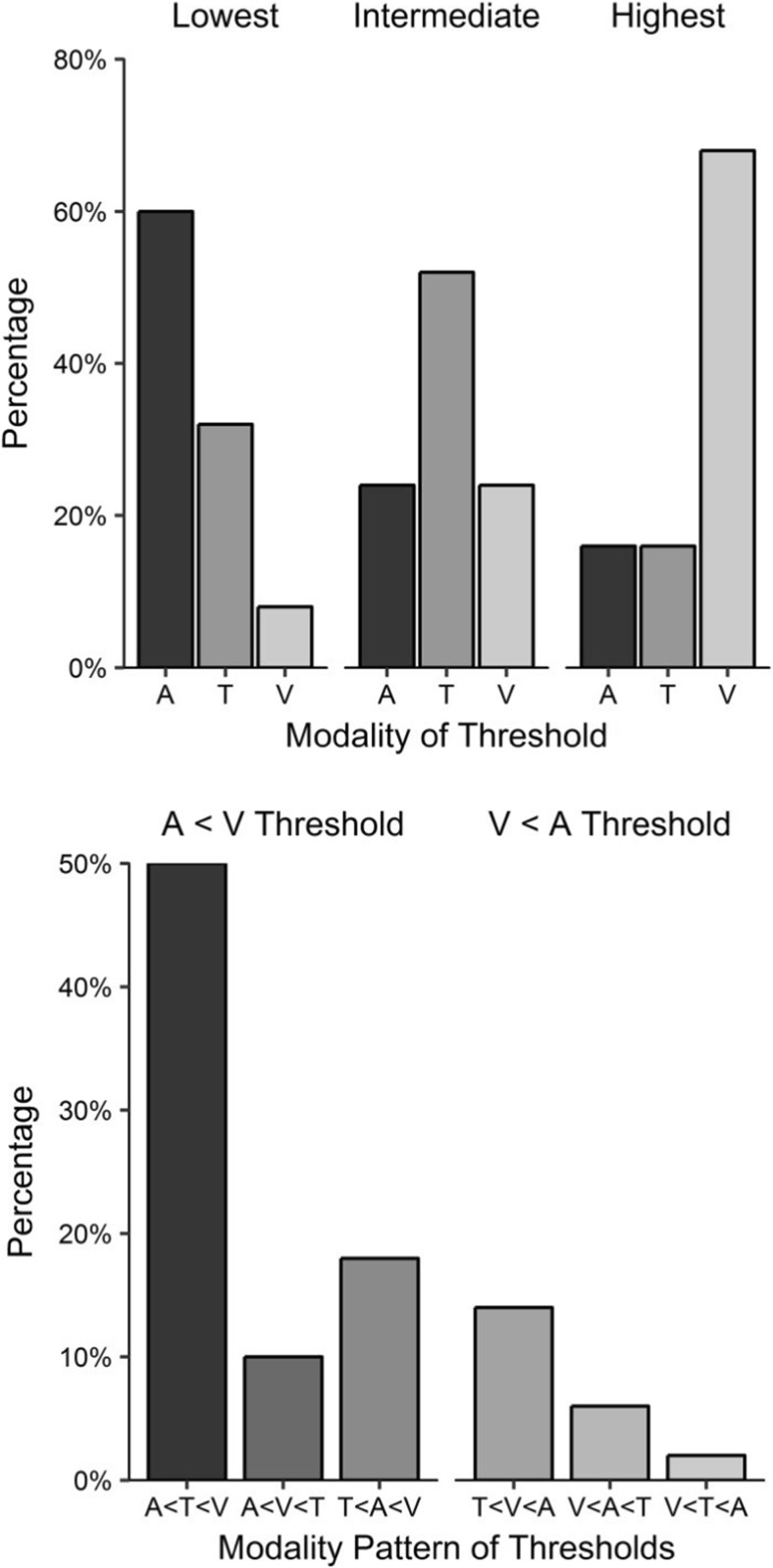
Inter-individual differences for Experiment 1A. (Upper panel) Percentages of each modality as participants’ lowest, intermediate, and highest thresholds; each cluster totals 100%. (Lower panel) Frequencies of each of the six possible modality patterns of thresholds. The left cluster indicates patterns with lower auditory than visual thresholds, and the right cluster indicates patterns with lower visual than auditory thresholds. A = Auditory, T = Tactile, and V = Visual intervals

We now consider the pervasiveness of modality differences by investigating the proportions of participants who achieved their lowest, intermediate, and highest thresholds in each of the modalities. We also investigate the most common *pattern* of modalities (lowest, intermediate, and highest in tandem). Two participants (P11 and P51) who were included in the previous analyses achieved the same threshold value for two modalities and so were excluded from this analysis.^12^ The upper panel of Fig. 3 shows the percentages of the remaining 50 participants whose lowest, intermediate, and highest thresholds were in each modality.

Most frequently, auditory intervals had the lowest threshold (60% of the time), tactile intervals had an intermediate threshold (52%), and visual intervals had the highest threshold (68%). The lower panel of Fig. 3 displays the percentages of participants whose modality ordering fell into each of the six possible patterns. The most frequent modality pattern was a lower auditory threshold, followed by an intermediate tactile threshold and a higher visual threshold (in 50% of cases). The next most frequent pattern was tactile, auditory, and then visual thresholds (18%). The least frequent pattern was visual (lowest), then tactile, then auditory (highest) thresholds, which was the case for one participant (2%). A total of 78% of participants had lower auditory than visual thresholds, whereas the remaining 22% had lower visual than auditory thresholds.

### Discussion

Thresholds for visual durations were significantly higher than those for auditory and tactile durations. In addition, auditory thresholds were significantly lower than tactile thresholds. Bayes factors favored the first two differences with extreme evidence, and with a moderate amount of evidence for the latter. Together, this confirms the classic effect and suggests that people have greater sensitivity to the durations of sounds and vibrations than they do for lights. This pattern of thresholds was reported previously by Jones et al. (2009), though they found no significant difference between tactile and visual thresholds.

To compare our findings with those of Jones and colleagues more precisely, Table 1 shows that the patterns of our standard deviations are the same—that is, smallest for auditory thresholds, intermediate for tactile thresholds, and highest for visual thresholds. Although the standard deviation of our visual thresholds appears to be quite larger than that of Jones and colleagues, the coefficient of variation (CV; i.e., *SD*/*M*) is relatively less large, and in fact is smaller than the CV for tactile thresholds. In addition, we replicated the finding that the largest CV was for auditory thresholds. We also replicated the finding of a greater standard error for visual (17.39 ms) than for auditory (9.75 ms) thresholds found in other work, such as the 800-ms standard condition in Experiment 1 of Grondin et al. (1998) and the 50-ms and 1,000-ms standard conditions of both Rammsayer, Buttkus, and Altenmüller (2012) and Rammsayer, Borter, and Troche (2015), though procedural and threshold calculation differences preclude direct comparisons of the values.

The exploration of inter-individual differences for Research Aim 2 revealed that the majority of participants’ lowest thresholds were for auditory intervals, their intermediate thresholds were for tactile intervals, and their highest thresholds were for visual intervals. Accordingly, exactly half of the participants’ thresholds followed this exact pattern. In addition, more than three-quarters of the participants had lower auditory than visual difference thresholds (78%). These descriptive differences suggest that, for most people, the pacemaker appears to run faster for auditory than for visual intervals, though the opposite is true for around one-fifth of people. It is worth bearing in mind that measurement error could have obscured the pattern of participants’ “true” pacemaker rates, but we present these incidences as a guide.

## Experiment 1B: Modality differences in verbal estimation

The threshold task revealed sensitivity to be similarly low for auditory and tactile intervals, and higher for visual intervals. If there is a relationship between thresholds and estimation slopes, as Jones et al. (2009) suggested, we would expect slopes to follow the same modality pattern as thresholds. Therefore, we now replicated Jones et al.’s estimation task, and predicted that slopes would be steepest for auditory intervals and shallowest for visual intervals. In addition, there might be no significant difference between the slopes for auditory and tactile intervals. We will consider inter-individual differences following the same steps as in Experiment 1A.

### Method

#### Participants

The same participants completed this experiment who had taken part in Experiment 1A.

##### Design

The first independent variable was the modality of the stimulus (auditory, tactile, or visual), and the second was its duration (77, 203, 348, 461, 582, 767, 834, 958, 1,065, or 1,183 ms).^13^ This short range of durations was used to prevent “chronometric counting” (Hinton & Rao, 2004; Wearden, 1991), in which participants might verbally or mentally measure time with a “one–one thousand, two–one thousand” type heuristic, which would reduce or negate the effects of presenting the stimuli in different modalities. The dependent variable was participants’ estimates of stimulus duration in milliseconds, which they typed into the keypad.

##### Apparatus and materials

The same apparatus and materials were used as in Experiment 1A.

##### Procedure

The task included three modality-specific blocks of 50 trials, presented in a random order, in which the ten stimulus durations were presented five times in each modality. Participants were told that the intervals were random durations between 50 and 1,250 ms and that they could only enter estimates within this range (inclusive). Trials were presented in a random order within each block. Each trial began with the presentation of a fixation cross for 500–1,000 ms, followed by the stimulus. Participants were prompted onscreen to type in their estimate in milliseconds and were reminded that 1 s = 1,000 ms. Five practice trials were presented at the start of each block, but advancement was not contingent on performance. The task was self-paced and took approximately 17 min to complete.

### Results

#### Verbal estimates

**Fig. 4:**
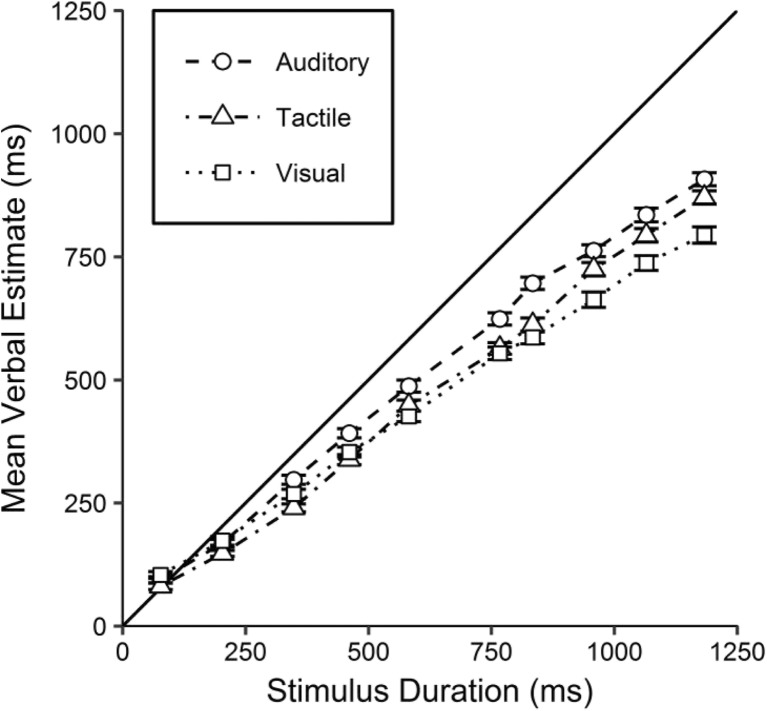
Mean verbal estimates for each modality against stimulus duration. The solid line represents perfectly veridical performance. Error bars indicate within-participants standard errors (Morey, 2008)

Participants who were unable to perform the task were excluded. This was defined as estimates being invariant as to stimulus duration, identified as linear functions not significantly different from the null when mean estimates for each duration were regressed against stimulus duration, separately for each participant. This led to the exclusion of one individual (P47),^14^ leaving a sample of 51 participants. See Fig. 4 for the mean verbal estimates for each modality across stimulus durations.

Inspection of Fig. 4 suggests a general underestimation of all except the shortest durations (77 ms) for all modalities. Estimates were higher for auditory durations, whereas tactile and visual estimates appear to be lower and quite similar, the same pattern found in Jones et al. (2009).

These suggestions were examined using a factorial ANOVA with two repeated measures factors: stimulus duration and modality. We found a significant main effect of stimulus duration, *F*(2.60, 130.17) = 750.70, *p* < .001, *η*_p_^2^ = .938. Post hoc analyses (Holm–Bonferroni corrected) revealed that each of the ten stimulus durations was estimated significantly differently from each of the others (*p* < .001 for all comparisons), which simply means that participants were sensitive to the presented duration.

There was also a significant main effect of modality, *F*(2, 100) = 7.50, *p* = .001, *η*_p_^2^ = .131. Post hoc analyses (Holm–Bonferroni corrected) revealed that participants estimated auditory durations to be significantly longer than both visual (*a* = .017, *p* = .002) and tactile (*a* = .025, *p* = .004) durations. However, the estimates for visual and tactile durations did not differ significantly (*a* = .050, *p* = .303).

The was also a significant Stimulus Duration × Modality interaction, *F*(8.39, 419.32) = 4.91, *p* < .001, *η*_p_^2^ = .089. This suggests that stimulus modality affected the slope of the function relating the mean estimates to stimulus duration, consistent with a multiplicative effect.

#### Slopes and intercepts

To investigate this interaction, we regressed each participant’s estimates against stimulus duration to extract slope and intercept values for each modality. See Table 2 for the resulting linear regression equations. The standard deviations of the auditory, tactile, and visual slope values were 0.18, 0.16, and 0.19, respectively, and the intercept values were 129.04, 91.84, and 118.21 ms.

**Table 2:** Mean slope and intercept values for each modality, extracted through linear regressions for each participant

Stimulus modality	Linear regression equation	
	Slope	Intercept
Auditory	Estimate = 0.76 ×	Stimulus Duration + 36.69 ms
Tactile	Estimate = 0.74 ×	Stimulus Duration + 4.97 ms
Visual	Estimate = 0.64 ×	Stimulus Duration + 52.90 ms

**Fig. 5:**
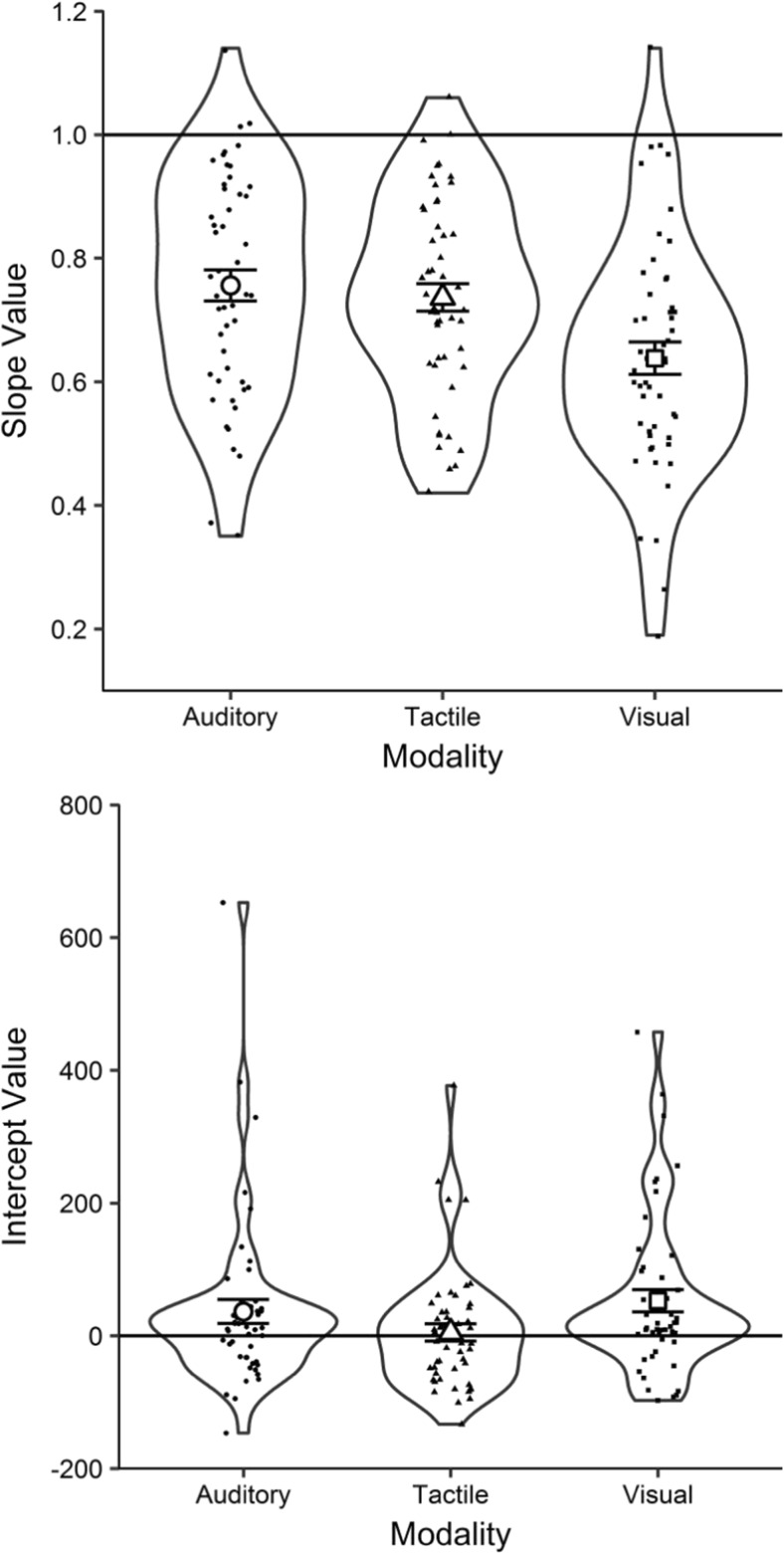
RDI plots (see Fig. 2 for a description), presenting the mean slope values (upper panel) and mean intercepts (lower panel) for auditory, tactile, and visual estimates. The solid lines represent perfectly veridical performance: a slope of 1 and an intercept of 0

Inspection of Table 2 suggests that the auditory and tactile functions have similar slopes but different intercepts—that is, they are approximately parallel. In contrast, the visual function appears to have a shallower slope than the others, but an intercept similar to that of the auditory function. The mean visual slope was 16% shallower than the mean auditory slope. See Fig. 5 for mean slopes and intercepts of these linear regressions.

Inspection of the upper panel of Fig. 5 suggests the slopes to be shallowest for visual estimates, and little difference between the slopes of auditory and tactile estimates. A repeated measures one-way ANOVA comparing the slopes across modalities revealed a significant difference between the three modalities, *F*(2, 100) = 12.76, *p* < .001, *η*_p_^2^ = .203. Post hoc analyses (Holm–Bonferroni corrected) confirmed that visual slopes were significantly shallower than both auditory (*a* = .017), *t*(50) = 4.33, *p* < .001, *BF*_–0_ = 548.74, *d* = 0.61, and tactile (*a* = .025), *t*(50) = 3.96, *p* < .001, *BF*_–0_ = 193.12, *d* = 0.56, slopes. Auditory and tactile slopes did not differ significantly (*a* = .050), *t*(50) = 0.86, *p* = .392, *BF*_0+_ = 2.96, *d* = 0.12.

Inspection of the lower panel of Fig. 5 suggests intercepts to be lowest for tactile estimates, followed by auditory estimates, and highest for visual estimates. A repeated measures one-way ANOVA was conducted to compare the intercepts of the estimation functions across modalities, and it revealed a significant difference between them, *F*(1.76, 87.75) = 6.86, *p* = .003, *η*_p_^2^ = .121.^15^ Post hoc analyses (Holm–Bonferroni corrected) confirmed that the intercepts for tactile durations were significantly lower than the intercepts for both visual (*a* = .017), *t*(50) = 3.40, *p* = .001, *BF*_–0_ = 43.96, *BF*_01_ = 0.05, *d* = 0.48, and auditory (*a* = .025), *t*(50) = 3.04, *p* = .011, *BF*_–0_ = 17.36, *BF*_01_ = 0.12, *d* = 0.43, durations. The intercepts for auditory and visual durations did not differ significantly (*a* = .050), *t*(50) = 1.11, *p* = .142, *BF*_01_ = 3.66, *d* = 0.16.

##### Research Aim 2: Exploration of inter-individual differences in auditory, tactile, and visual slopes

**Fig. 6:**
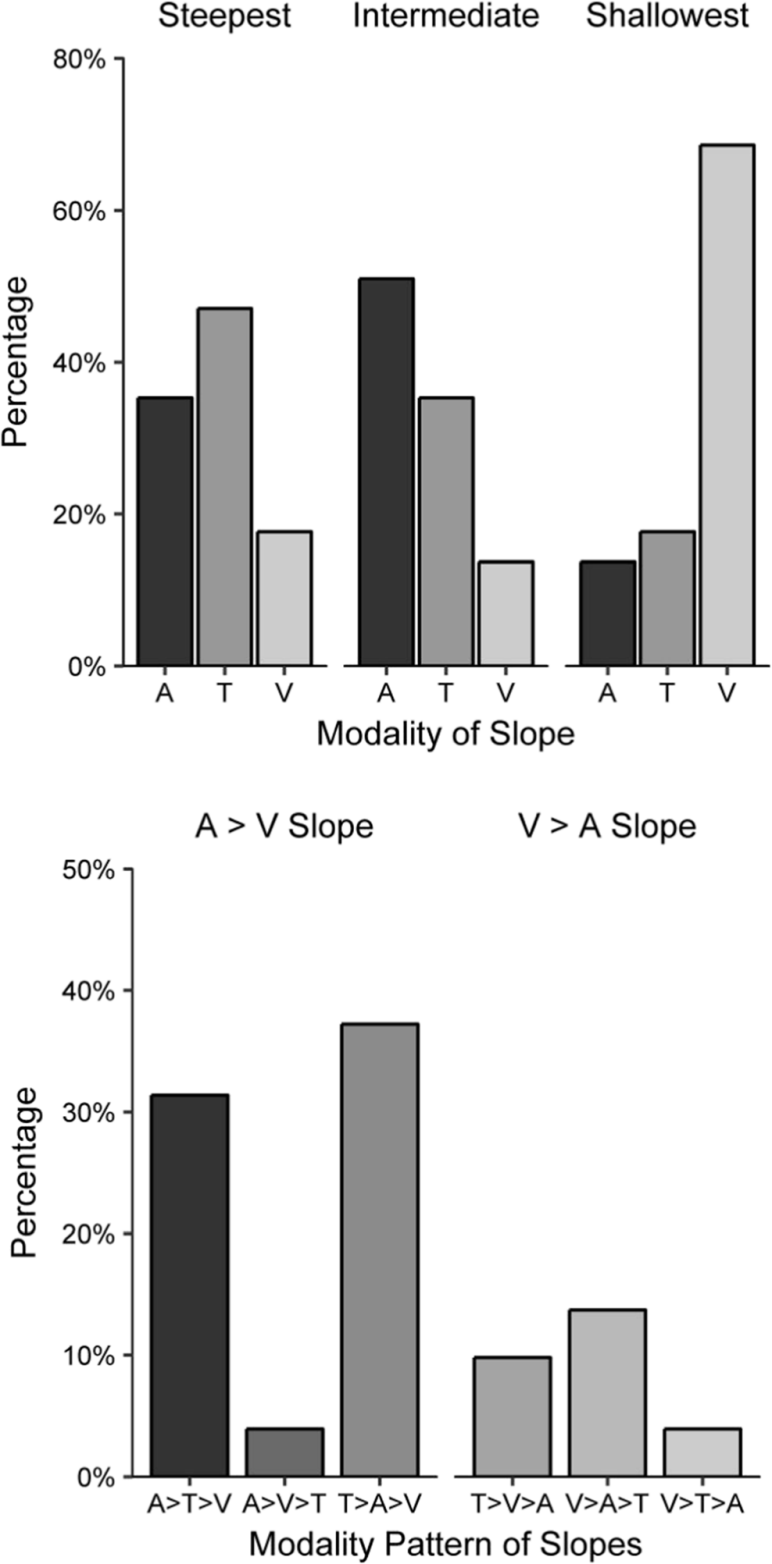
Inter-individual differences in Experiment 1B. (Upper panel) Percentages of each modality as participants’ steepest, intermediate and shallowest slopes; each cluster totals 100%. (Lower panel) Frequencies of each of the six possible modality patterns of slopes. The left cluster indicates patterns with steeper auditory than visual slopes, and the right cluster indicates patterns with steeper visual than auditory slopes. A = Auditory, T = Tactile, and V = Visual intervals

The upper panel of Fig. 6 presents the percentages of the 51 participants^16^ whose steepest, intermediate, and shallowest slopes fell within each of the modality categories. No participants achieved the same slope value in two or more modalities.

The most frequent steepest slope was for not for auditory intervals, but for tactile intervals (47%). The most frequent intermediate slope was for auditory intervals (51%), and the most frequent shallowest slope was for visual intervals (69%). The lower panel of Fig. 6 displays the percentages of participants whose modality orders corresponded to each of the six possible patterns. The most frequent modality pattern was a steeper tactile slope, followed by an intermediate auditory slope and a shallower visual slope (37%). The next most frequent pattern was auditory, then tactile and visual slopes (31%). The least frequent patterns were auditory, then visual and tactile slopes (4%) and visual, then tactile and auditory slopes (4%), with two participants apiece. A total of 73% of the participants had steeper auditory than visual slopes, whereas the remaining 27% had steeper visual than auditory slopes.

### Discussion

As had been found in previous research, durations in all three modalities were generally underestimated (Jones et al., 2009; Wearden et al., 1998). The initial factorial ANOVA showed durations to be estimated as relatively longer (i.e., underestimated less) when presented as auditory intervals than when presented as tactile or visual intervals of the same physical duration. Estimates for tactile and visual durations were found not to differ significantly. In terms of our slope analyses, the slopes for visual estimates were significantly shallower than those for auditory and tactile estimates, indicating an apparent difference in pacemaker rate. However, auditory slopes were not significantly higher than tactile slopes.

Although the significant difference between auditory and visual slopes found in our data is well-reported (Jones et al., 2009; Wearden et al., 1998, Wearden et al., 2006), Jones and colleagues found that tactile slopes did not differ from visual slopes. Therefore, although the evidence suggesting a difference in pacemaker rates for auditory and visual intervals is robust, it is not clear whether pacemaker rates for tactile intervals are as distinct.

Although we were able to compare the standard deviations of our thresholds to those of Jones and colleagues, these authors did not provide the standard deviations of their slope values. In the absence of this direct comparison, comparison of our standard errors to those presented in their Fig. 3 (upper panel) suggests that we replicated the effect of the smallest standard error for tactile intervals (0.022, in our case). However, we found the highest standard error for visual slopes (0.026), and an intermediate standard error for auditory slopes (0.025), whereas Jones et al. (2009) found the opposite pattern (though our difference was marginal).

In addition to slope differences, there were also some significant differences in intercepts. Intercepts were significantly lower for tactile estimates than for auditory and visual estimates. Scalar timing theory interprets intercept differences as differences in switch latency (Jones et al., 2009; Wearden et al., 1998). Therefore, this suggests that the switch has a reduced latency when timing tactile intervals than in the other two modalities. This contrasts with Jones et al.’s finding of no significant differences in intercepts.

The exploration of inter-individual differences for Research Aim 2 showed that steeper tactile slopes were more common than steeper auditory slopes. This might seem at odds with the mean auditory slope being higher in value than the mean tactile slope, but the ranking of slopes does not take magnitude differences into account. It may have been the case that one group of participants had slightly steeper tactile than auditory slopes, whereas another group had much steeper auditory than tactile slopes. More than two-thirds of participants had their shallowest slope for visual intervals. When looking at the overall pattern of slopes, the most frequent one (37%) was for participants to have steeper tactile, intermediate auditory, and shallower visual slopes. This was closely followed by participants who had steeper auditory, intermediate tactile, and shallower visual slopes (31%). Therefore, though the auditory–tactile–visual pattern manifested in the mean data, this pattern was not the most common at the individual level. Regardless of the placement of tactile intervals, around three-quarters of participants had steeper auditory than visual slopes (73%). This suggests that the pacemaker may run faster for auditory than for visual intervals for most people, but the opposite may be true for around a quarter of people. We do, however, acknowledge the role of measurement error, which may have obscured the pattern of participants’ true pacemaker rates, and we will take this into consideration in later discussions. Finally, the pacemaker rate resulting from tactile intervals is not as clear.

## Research Aim 3: Exploration of intra-individual differences in auditory, tactile, and visual thresholds and slopes

Since it has been argued that performance in estimation and threshold tasks in both cases is determined by pacemaker rate (Jones et al., 2009), we would expect negative correlations between performance in each task. This is because a faster pacemaker would lead to more pulses being produced during a certain period equating to multiplicatively higher estimates (Jones et al., 2009; Wearden et al., 1998). In addition, this higher resolution of pulses has been argued to lead to higher accuracy and sensitivity, as indicated by lower temporal difference thresholds (Rammsayer, 2008; Rammsayer & Grondin, 2000; Troche & Rammsayer, 2011).

Since the temporal difference thresholds in our experiments relate to a standard duration of 700 ms, a more direct comparison of the effects of pacemaker rate in the two tasks would compare thresholds and participants’ predicted estimates of 700 ms. We therefore used each participant’s linear regression equation relating estimated duration to stimulus duration to predict their personal estimates of 700 ms. This correlation was also conducted within each modality.

It could be argued that the two tasks place quite different demands on participants, in that estimation requires greater higher-order processing than duration discrimination, and is also subject to quantization. Correlations can also be affected by limited variability in the two variables and by differences in the shapes of the distributions (Goodwin & Leech, 2006). Therefore, even if the pacemaker account of both tasks is correct, correlations might not be found for these reasons. In an attempt to sidestep these issues and consider the assumption from another angle, a more coarse-grained comparison of the two tasks was conducted, by treating the modalities of slopes and intercepts as ordinal data. Suppose that it is true for a certain participant that their pacemaker runs faster for auditory than for tactile intervals, and for tactile than for visual intervals. If a task-independent pacemaker is responsible for determining thresholds and slopes, then in both tasks this participant should probably perform best with auditory intervals, intermediate with tactile intervals, and worst with visual intervals. That is, a participant’s best performance should be in a congruent (i.e., the same) modality across tasks, if performance is determined in both cases by pacemaker rate. The same could be said for participants’ intermediate and worst performance. Therefore, if the pacemaker explanation is correct, we would expect to see this congruency effect for most participants.^17^

### Results

#### Research Aim 3A: Correlational analyses

**Fig. 7:**
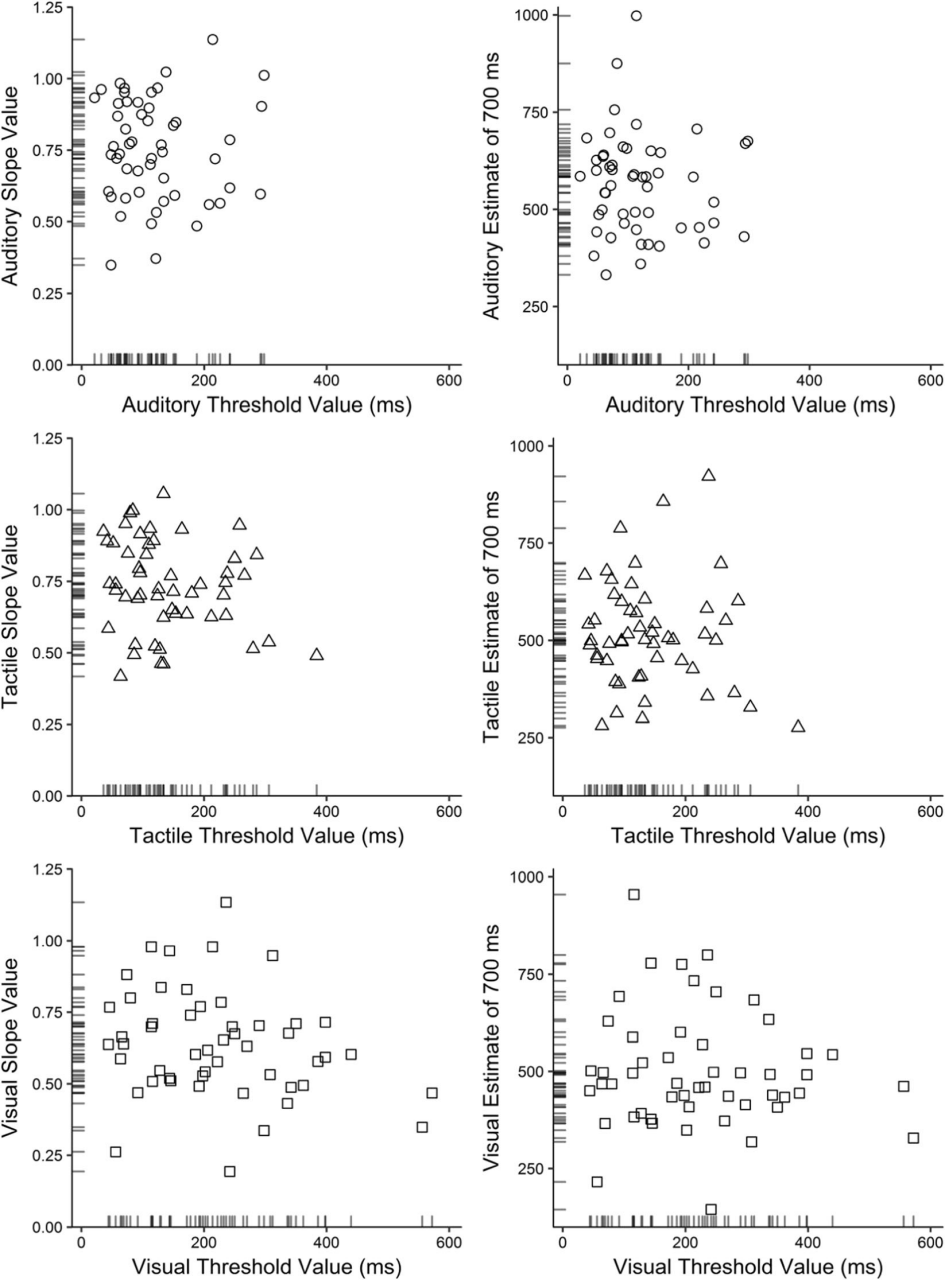
Scatterplots between temporal difference thresholds and estimation slopes (left column), and between thresholds and predicted estimates of 700 ms (right column) in Experiment 1. The internal axis markers indicate the density of the data

The same participant who was excluded from Experiment 1B was excluded from the relevant correlations. Pearson’s product-moment correlations (Holm–Bonferroni corrected) were conducted to compare thresholds with the estimation slopes and predicted estimates of 700 ms within each modality.^18^ However, no significant correlations were found within any of the three modalities (see Table 3). Figure 7 shows scatterplots for each correlation, where the position of each panel relates to the location of the respective test in Table 3.

**Table 3:** Pearson correlations between temporal difference thresholds and both estimation slopes and simulated 700-ms estimates

Threshold	Estimation slope					Predicted estimate for 700 ms				
	*n*	*r*	*α*	*p*	*BF*_0–_	*n*	*r*	*α*	*p*	*BF*_0–_
Auditory	52	– .017	.050	.907	5.26	52	– .077	.025	.590	3.58
Tactile	52	– .207	.025	.142	1.09	52	– .093	.050	.511	3.16
Visual	51	– .237	.025	.094	0.77	51	– .098	.050	.494	3.05

*α* relates to the alpha criterion (Holm–Bonferroni corrected, which was applied across each row), and Bayes factors express the amount of evidence in favor of the data given the null hypotheses. See the supplementary material for nonparametric tests confirming these results

Likewise, one-tailed Bayes factors favored the null hypotheses over the alternative hypotheses with anecdotal to moderate evidence, with one exception: Anecdotal evidence was found in support of the hypothesis of a negative correlation between thresholds and slopes for visual intervals, with the data being 1.3 times more likely under this hypothesis than the null.

#### Research Aim 3B: Ordinal analyses

**Fig. 8:**
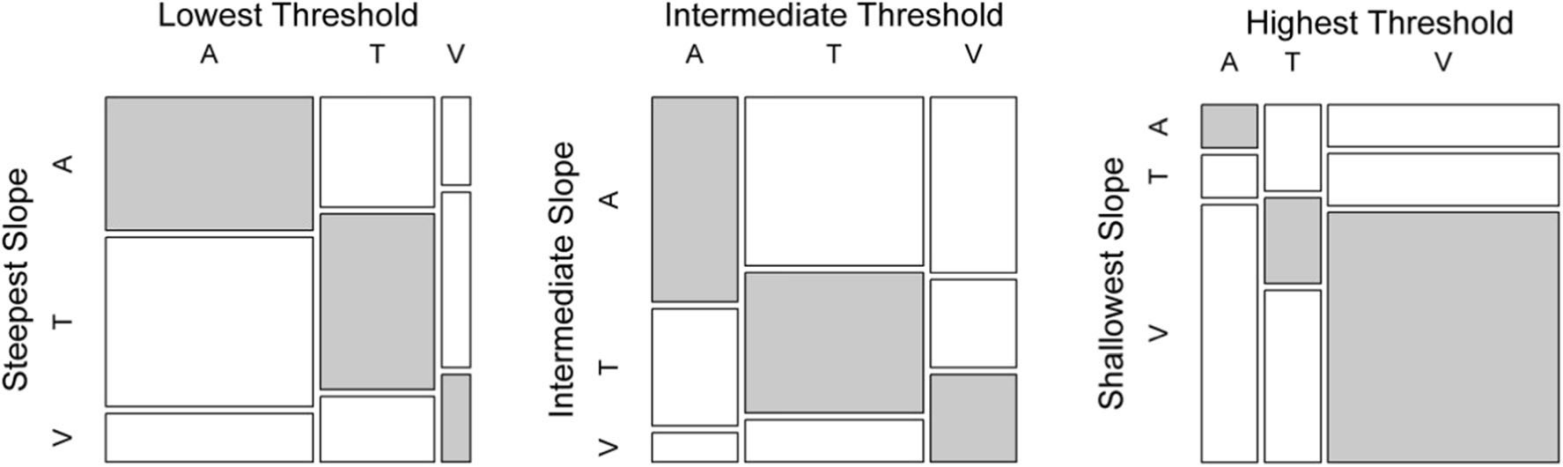
Mosaic plots representing the frequency of each modality for highest pacemaker rates (left), intermediate pacemaker rates (center), and lowest pacemaker rates (right). The area of each tile indicates the proportion of participants who fall into each of the nine slope-threshold combinations. Shading represents the tiles in which the modalities of slopes are congruent. A = Auditory, T = Tactile, and V = Visual intervals

The same three participants who were excluded from the inter-individual differences sections (Research Aim 2) of Experiments 1A and 1B^19^ were also excluded from this analysis, leaving a total of 49 participants. The left panel of Fig. 8 brings together the leftmost clusters of Fig. 3 (upper panel) and Fig. 6 (upper panel), combining each one-dimensional bar chart into a two-dimensional mosaic plot. The center and right panels here likewise bring together data in the central and right clusters in the earlier figures. The benefit of this combination is the ability to see the relationship between thresholds and slopes at the individual level, without the effect of magnitude differences, which may have obscured the correlations. This allows us to assess whether participants whose lowest threshold was for auditory intervals, for example, also had their highest slope for auditory intervals.

Note that the aim of this analysis is not to compare the observed proportions of participants to chance. We are not testing a null hypothesis of “no association” between the thresholds and slopes, for which a chi-square test would be appropriate. Rather, we simply aim to examine the prediction that most participants’ best performance should be in a congruent (i.e., the same) modality across tasks, for which there is no relevant significance test, to our knowledge. We do not expect this to occur for *all* participants, since it is likely that some measurement error could be present that may mask a congruency effect in some participants. Rather, we will interpret a congruency effect as present if more than half of the participants’ best (or intermediate, or worst) performance occurs for the same modality.

A total of 41% of participants achieved their steepest slopes and lowest thresholds in the same modality, whereas 59% of participants did not. Coincidentally, a total of 41% of participants achieved their intermediate thresholds and slopes in the same modality, whereas the remaining participants did not. Finally, 55% of participants had the same modality for their shallowest slopes and highest thresholds (49% for visual intervals), whereas 45% did not have these in the same modality. Therefore, it was more common for participants to achieve their best (and intermediate) performance in each task in incongruent modalities, whereas most participants performed worst in each task in the same modality.

### Discussion

It has been argued that performance in threshold and estimation tasks are largely determined by the pacemaker, which runs at a faster rate for auditory than visual intervals (Jones et al., 2009; Wearden et al., 1998). Therefore, if pacemaker rate is consistent across tasks, and if performance in both tasks is determined largely by pacemaker rate, the thresholds and estimates within each modality should correlate. However, we found no significant correlations between thresholds and estimates within any modality using either slopes or predicted estimates of 700 ms. Additionally, although one Bayes factor (for visual thresholds and slopes) anecdotally favored the alternative hypothesis, the remaining five supported the data given the null hypothesis with anecdotal to moderate evidence. Taken together, the lack of correlations between thresholds and slopes (and predicted estimates of 700 ms) suggests that performance on one or both of these tasks is not wholly determined by pacemaker rate, contrary to the suggestion of Jones et al. (2009). It could be argued that estimates (magnitude judgments) and thresholds (discrimination judgments) rely on different mechanisms and are of different levels of abstraction. However, this was an initial and simple test of the application of the pacemaker explanation to both tasks.

Being mindful of the factors that could mask correlations (task differences, variability of measures, and differences in distribution shapes), we investigated the same general question from another angle, using a more coarse-grained approach. Here we evaluated the implicit assumption that, at the individual level, the modality in which pacemaker rate is highest (or intermediate, or lowest) should generally be the same in each task. We found that it was more common for participants to achieve their best performance in *different* modalities in each task (59%). The same was true for participants’ intermediate performance (59%). However, the assertion proved true for the lowest pacemaker rates, for which more than half of the participants (55%) achieved their shallowest slope and highest threshold in the same modality. We allowed for the possible role of measurement error by interpreting the presence of a congruency effect if more than half of the participants (not *all* of the participants) performed best (or intermediate, or worst) in the same condition in each task. The lack of a consistent congruency effect, therefore, suggests that it may be too simple an explanation that a putative pacemaker is a strong determiner of performance in one or both of these tasks.

On the other hand, it may be the case that the inherent task differences were too great to allow us to find relationships using correlations and ordinal intra-individual differences. These task differences are highlighted by the differential effects of stimulus modality on thresholds and slopes; visual intervals led to slopes 16% shallower than auditory slopes, and thresholds 85% higher than those for auditory intervals, on average. This was reflected in a higher effect size for auditory–visual differences in thresholds (*d* = 0.90) than in slopes (*d* = 0.61). Furthermore, the exploration of intra-individual differences with a focus on auditory–visual may have been obfuscated through the inclusion of tactile intervals in this analysis. Although it was principled to include tactile thresholds and slopes (especially when investigating intra-individual differences), they did not differ significantly from auditory thresholds and slopes. This suggests that in our data, at least, tactile performance did not lead to a robust change in pacemaker rates. This presents further motivation for investigating the same research aims with a larger and more robust effect: the filled-duration illusion.

## Experiment 2A: Filled–empty differences in temporal difference thresholds

In the following sections we describe experiments and analyses identical to those in Experiment 1, this time using filled and empty intervals. Following previous research (e.g., Rammsayer, 2010), we expected that thresholds for filled intervals would be significantly lower than those for empty intervals. Inter-individual differences in estimates of filled and empty intervals had been investigated by Hasuo et al. (2014), whose cluster analysis revealed two subgroups of participants. Cluster 1 (64%) displayed little difference between estimates of filled and empty intervals, whereas Cluster 2 (36%) exhibited clear overestimations of the duration of filled as compared to empty intervals. We considered inter-individual differences for Research Aim 2 in the same manner as in Experiments 1A and 1B.

### Method

#### Participants

Thirty-two participants^20^ (students of the University of Manchester) completed Experiments 2A and 2B in a random order and received £5 for their time.

#### Design

The independent variable was the type of the stimulus (filled or empty), and the dependent variable was participants’ resulting temporal difference thresholds.

#### Apparatus and materials

The same apparatus and materials were used as in Experiments 1A and 1B, with the exception of the chin rest, foam grip, and ear protectors. The empty stimuli were durations of silence—for example, for 700 ms—delineated by 25-ms, 500-Hz tones immediately before and after this duration. The auditory stimuli of Experiments 1A and 1B were repurposed as the filled stimuli.

#### Procedure

Participants completed a 50-trial adaptive threshold task for both stimulus types in a random order. The procedure followed that of Experiment 1A. This experiment took approximately 12 min to complete.

### Results

#### Temporal difference thresholds

**Fig. 9:**
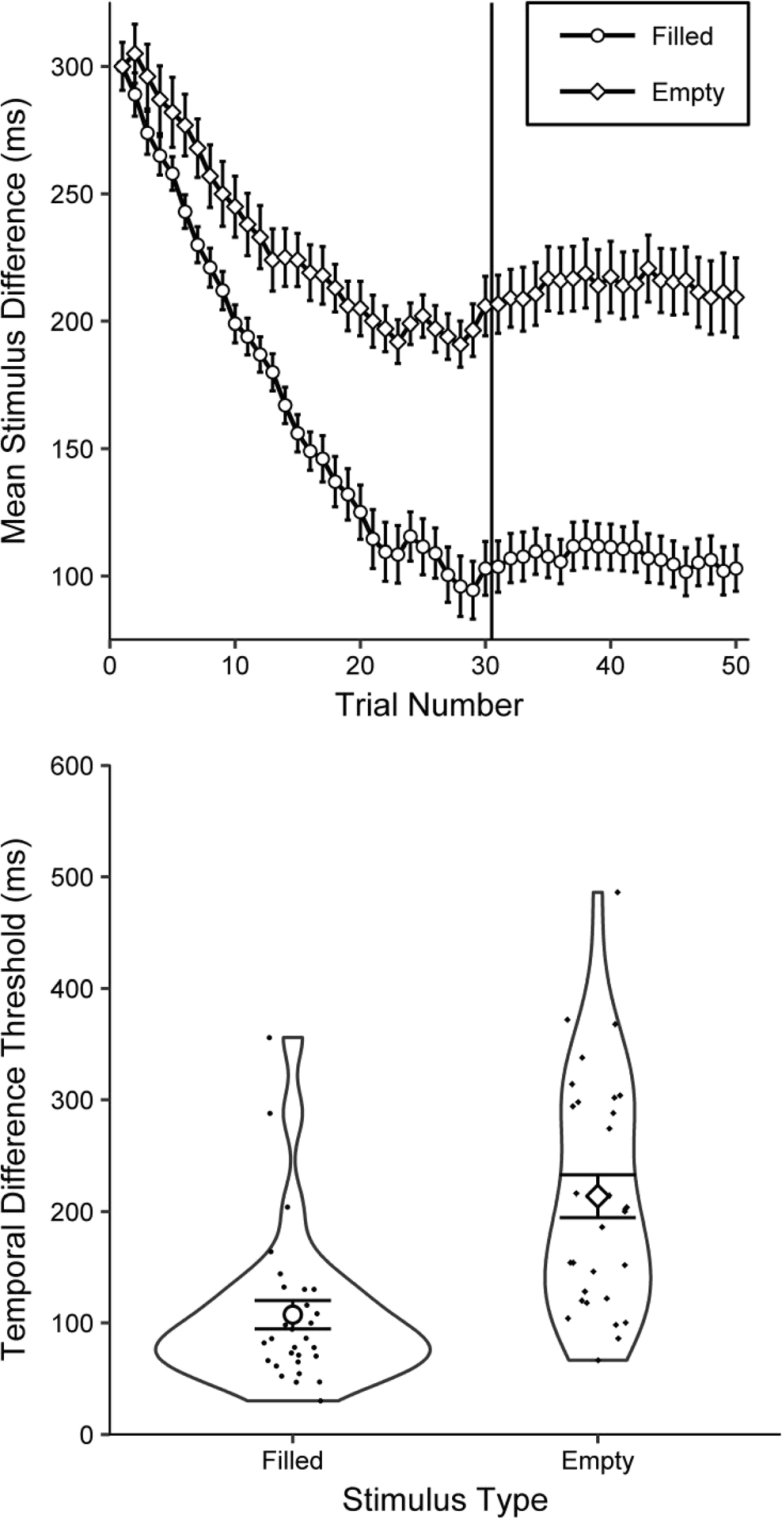
Threshold performance for Experiment 2A. (Upper panel) Mean differences between the standard and comparator across the 50 trials for each stimulus type. The vertical solid line separates the last 20 trials, over which the step size was reduced and the temporal difference thresholds were calculated. Error bars denote within-participants standard errors (Morey, 2008). (Lower panel) RDI plots (see Fig. 2 for a description) presenting the mean temporal difference thresholds for filled and empty intervals

Outliers were defined once more as thresholds greater than 600 ms, which led to the exclusion of two individuals (P56 and P58),^21^ leaving a sample of 30 participants. The upper panel of Fig. 9 shows the mean differences between the standard and comparator durations across the 50 trials for filled and empty durations.

Inspection of the upper panel of Fig. 9 suggests that participants found the task easier for filled than for empty durations, as indicated by a sharper decline in the difference in duration between the standard and the comparator, as well as by the lower within-participant standard errors. Note that, on average, participants answered the first empty trial incorrectly, as demonstrated by the second diamond being higher than the first. The lower panel of Fig. 9 presents the resulting temporal difference thresholds. Inspection suggests lower thresholds for filled than for empty intervals, which was confirmed with a paired-samples *t* test, *t*(29) = 6.50, *p* < .001, *BF*_–0_ = 81,155, *d* = 1.19.^22^ The mean threshold for empty intervals (213.62 ms) was 99% higher than the mean threshold for filled intervals (107.33 ms). The standard deviations were 104.04 and 70.07 ms, respectively.

#### Research Aim 2: Exploration of inter-individual differences in filled and empty thresholds

A total of 93% of the 30 participants had a lower threshold for filled than for empty intervals. Only two participants showed the opposite pattern, and no participants achieved the same threshold value for both filled and empty intervals.

### Discussion

Temporal difference thresholds for filled durations were significantly lower than those for empty durations, as had previously been found by Rammsayer (2010), among others. This suggests that people have greater sensitivity to the durations of continuous tones than they have for periods of silence delineated by two short beeps. This is consistent with the idea that the pacemaker runs faster for filled than for empty intervals. Although we cannot directly compare our standard deviations to those of other research due to differences in procedure and the calculation of thresholds (e.g., using Weber fractions), we replicated the effect of a greater standard deviation for the temporal sensitivity to empty than to filled intervals (e.g., the 50-ms and 1,000-ms standard conditions in Exp. 1 of Rammsayer, 2010).

The exploration of inter-individual differences for Research Aim 2 revealed that almost all participants (93%) had lower thresholds for filled than for empty intervals. This suggests that it may indeed be true that the pacemaker runs faster for filled than for empty intervals for most people.

## Experiment 2B: Filled–empty differences in verbal estimation

Wearden et al. (2007) investigated the filled-duration illusion using the verbal estimation task and found that slopes for empty intervals were approximately 35% shallower than those for filled intervals. We aimed to replicate this effect, then to examine the relationship between the thresholds and slopes for filled and empty intervals as Research Aim 3.

### Method

#### Participants

The same participants completed this experiment who had taken part in Experiment 2A.

#### Design

The first independent variable was the type of stimulus (filled or empty), and the second independent variable was the duration of the stimulus (77, 203, 348, 461, 582, 767, 834, 958, 1,065, or 1,183 ms), as had been done in Experiment 1B. The dependent variable was a participant’s verbal estimation of the stimulus duration in milliseconds, which was typed into the keypad.

#### Apparatus and materials

The same apparatus and materials were used as in Experiment 2A.

#### Procedure

This task followed the same procedure as in Experiment 1B and took approximately 11 min to complete.

### Results

#### Verbal estimates

**Fig. 10:**
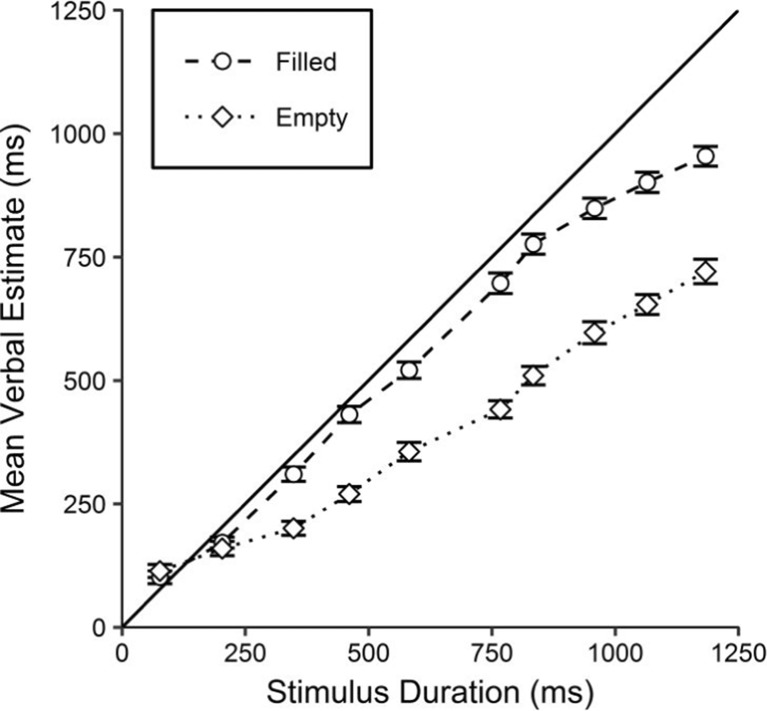
Mean verbal estimates for filled and empty intervals, against the stimulus durations. The solid line represents perfectly veridical performance. Error bars indicate within-participants standard errors (Morey, 2008)

Participants who were unable to perform the task were excluded, as in Experiment 1B. This led to the exclusion of two individuals (P58 and P64),^23^ leaving a sample of 30 participants. See Fig. 10 for the mean verbal estimates for filled and empty intervals.

Inspection of Fig. 10 suggests a general underestimation of all durations except the shortest (77 ms) for both stimulus types. Estimates were higher for filled durations, whereas empty durations appear to have been judged as multiplicatively shorter.^24^

The hypothesis that verbal estimates would be higher for filled than for empty durations was examined using a factorial ANOVA with two repeated measures factors: stimulus duration and stimulus type. The ANOVA found a main effect of stimulus duration, *F*(2.62, 75.91) = 217.67, *p* < .001, *η*_p_^2^ = .992. There was also a main effect of stimulus type, *F*(1, 29) = 17.00, *p* < .001, *η*_p_^2^ = .370, in which the estimates of empty durations were significantly shorter than those of filled durations. The interaction between stimulus duration and stimulus type was also significant, *F*(3.90, 113.00) = 11.31, *p* < .001*, η*_p_^2^ = .281, suggesting a multiplicative effect.

#### Slopes and intercepts

Slope and intercept values were extracted for each participant in the same manner as in Experiment 1B. See Table 4 for the resulting linear regression equations. The standard deviations of the filled and empty slope values were 0.24 and 0.25, respectively, and for the intercept values, 143.83 and 135.73 ms.

**Table 4:** Mean slope and intercept values for each modality, extracted through linear regressions for each participant

Stimulus type	Linear regression equation	
	Slope	Intercept
Filled	Estimate = 0.82×	Stimulus Duration + 38.03 ms
Empty	Estimate = 0.57×	Stimulus Duration + 31.12 ms

**Fig. 11:**
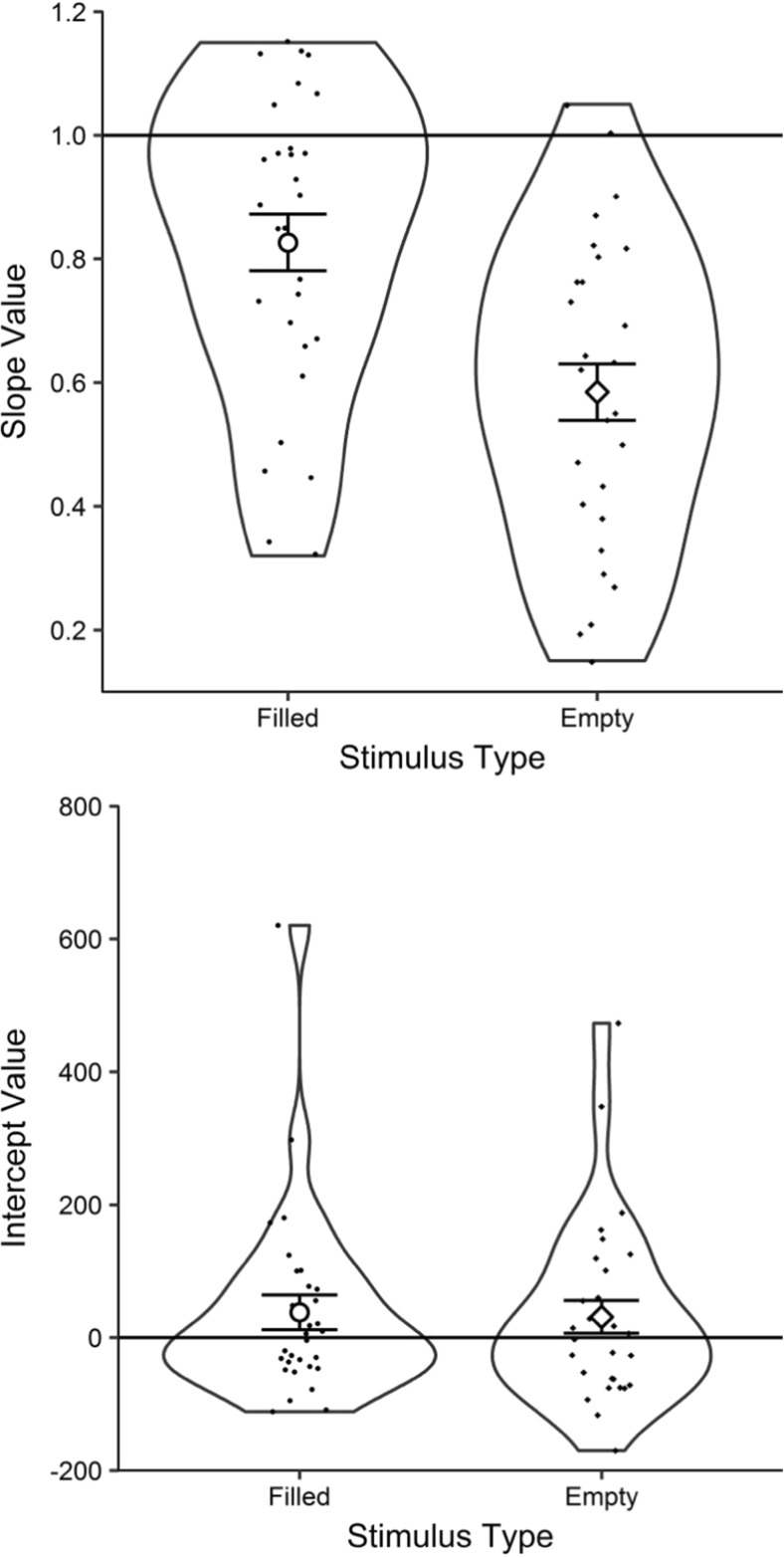
RDI plots (see Fig. 2 for a description) presenting the mean slope values (upper panel) and mean intercepts (lower panel) for filled and empty estimates. The solid lines represent perfectly veridical performance: a slope of 1 and an intercept of 0

Inspection of Table 4 suggests that the estimates for filled and empty durations differ in slope, with little difference in the intercepts. The mean empty slope was 30% shallower than the mean filled slope. See Fig. 11 for the mean slopes and intercepts of these linear regressions.

The upper panel of Fig. 11 suggests that slopes were shallower for empty than for filled intervals. A paired-samples *t* test confirmed this difference, *t*(29) = 4.65, *p* < .001, *BF*_–0_ = 742.73, *d* = 0.85.

The lower panel of Fig. 11 suggests no difference in intercepts, which was confirmed by a paired-samples *t* test, *t*(29) = 0.21, *p* = .835, *BF*_01_ = 5.04, *d* = 0.04.^25^

#### Research Aim 2: Exploration of inter-individual differences in filled and empty slopes

In all, 83% of the 30 participants^26^ had a steeper slope for filled than for empty intervals. Only four participants showed the opposite pattern, and none achieved the same slope value for filled and empty intervals.

### Discussion

The initial factorial ANOVA showed that durations were estimated as relatively longer when presented as filled than when presented as empty intervals. Further analysis showed the slopes for empty intervals to be shallower than those for filled intervals. This is consistent with the idea that the pacemaker runs faster for filled than for empty intervals, and confirms the findings of previous research (Wearden et al., 2007). However, the omission of a measure of slope variability from the earlier authors precludes a comparison of our standard deviations, because to our knowledge no other published study has compared the estimation slopes for filled and empty intervals. The intercepts were found not to differ, suggesting that both stimulus types have similar switch latencies. This finding was in contrast to that of Wearden et al. (2007), who found a significantly higher intercept for filled than for empty intervals.

The exploration of inter-individual differences for Research Aim 2 revealed that steeper slopes for filled intervals (83%) were more common than steeper slopes for empty intervals. This suggests that the pacemaker may run faster for filled than for empty intervals for most people, though the opposite may be true for around 17% of people. However, it is possible that the underlying pacemaker rates could be obscured here by measurement error. Nevertheless, this presents a clearer picture than the inter-individual differences found Experiment 1B, and in turn it may suggest a higher possibility of finding correlations within each condition in Research Aim 3.

## Research Aim 3: Exploration of intra-individual differences in filled and empty thresholds and slopes

Following the same line of investigation as with modality differences, we considered correlations between thresholds and both slopes and the predicted estimates of 700 ms. Following the description of these correlations, we will explore ordinal intra-individual differences in each task, in an attempt to sidestep task differences between estimations and thresholds, as well as other issues with correlational analyses mentioned earlier.

### Results

#### Research Aim 3A: Correlational analyses

**Fig. 12:**
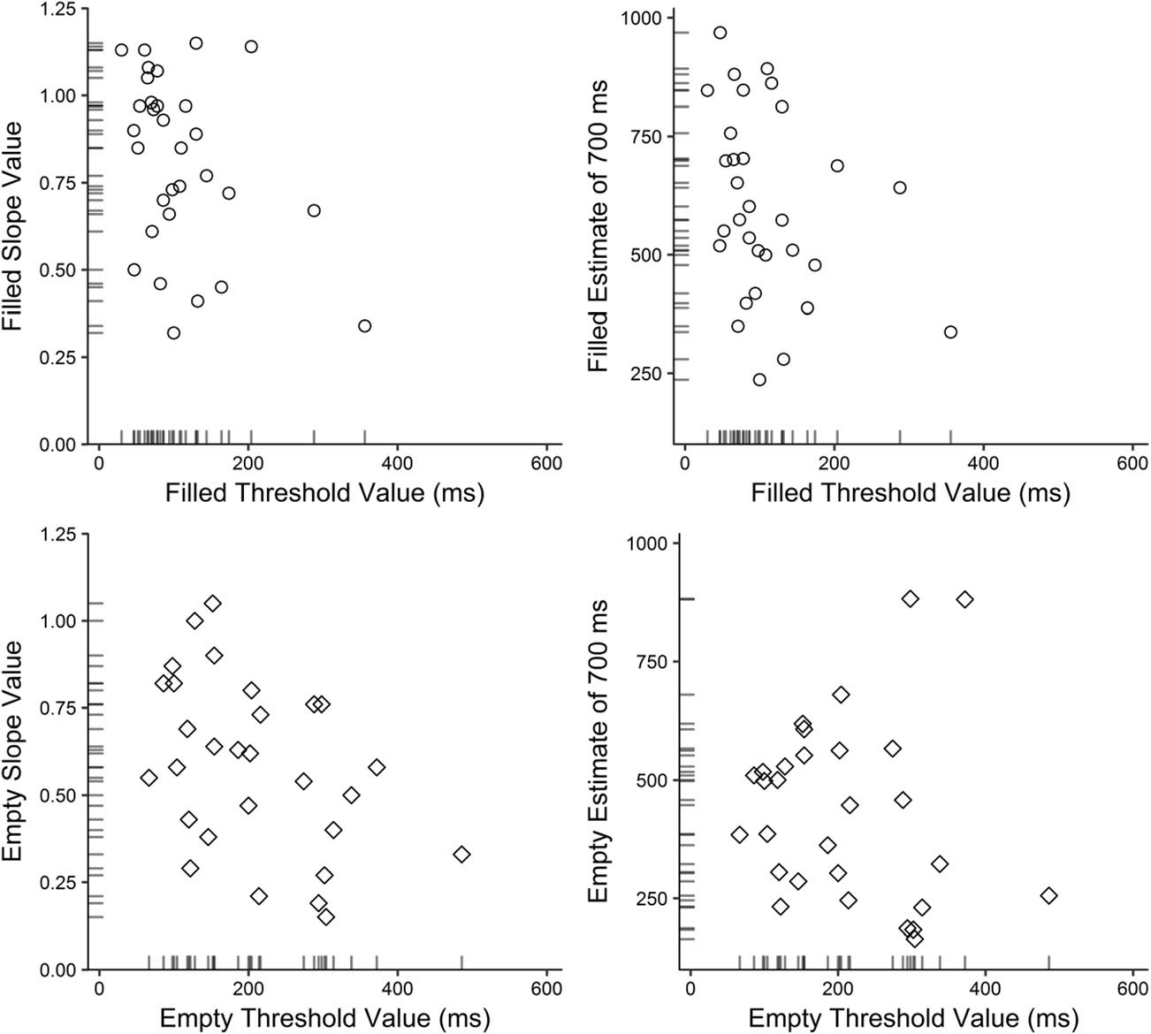
Scatterplots between temporal difference thresholds and estimation slopes (left column), and thresholds and predicted estimates of 700 ms (right column) in Experiment 2. Internal axis markers indicate the density of the data

The same participants who were excluded from Experiments 2A and 2B were excluded from the relevant correlations. Pearson’s product-moment correlations (Holm–Bonferroni corrected) were conducted between thresholds and both estimation slopes and predicted estimates of 700 ms, for filled and empty intervals (see Table 5). Figure 12 shows scatterplots for each type of correlation, where the position of each panel relates to the location of the respective test in Table 5.

**Table 5:** Pearson correlations between temporal difference thresholds and both estimation slopes, and predicted estimates of 700 ms in Experiment 2

Threshold	Estimation slope					Predicted estimate of 700 ms				
	*n*	*r*	*α*	*p*	*BF*_–0_	*n*	*r*	*α*	*p*	*BF*_–0_
Filled	31	– .377	.025	.037	3.51	31	– .306	.050	.094	1.62
Empty	29	– .431	.025	.020*	6.12	29	– .087	.050	.655	0.34

*α* relates to the alpha criterion (Holm–Bonferroni correction, applied to each row), and * indicates significance. Bayes factors express the amount of evidence in favor of the data given the alternative hypotheses. See the supplementary material for nonparametric tests confirming these results

Considering performance for filled intervals, the correlations were not significant, either between thresholds and estimation slopes or between thresholds and predicted estimates. However, Bayes factors favored the data given a negative correlation for each, with moderate and anecdotal evidence, respectively. A moderate negative correlation was found between thresholds and slopes for empty intervals, whereas the correlation between empty thresholds and predicted estimates was not significant. These results were supported by their accompanying Bayes factors, with moderate and anecdotal evidence, respectively. This suggests that the better a person’s discrimination threshold for empty intervals, the steeper their estimation slope for such intervals.

#### Research Aim 3B: Ordinal analyses

The same participants who were excluded from Experiments 2A and 2B^27^ were also excluded from this analysis, leaving a total of 29 participants. Most participants’ steepest slopes and lowest thresholds were for filled intervals (79%). Empty intervals led to four participants’ (14%) steepest slopes and to two participants’ (7%) lowest thresholds. No participants in our sample had both a steeper slope and a lower threshold for empty than for filled intervals.

### Discussion

Once again, we sought to test the assertion that filled–empty differences in thresholds and estimation slopes are due to differences in pacemaker rate. Out of the four correlations, one was found to be significant, where lower thresholds for empty intervals relate to steeper slopes for empty intervals (and vice versa). This suggests that the ability to discriminate between differences in the duration of empty intervals is linked to the estimation of the duration of empty intervals. However, the same correlation was not significant for filled intervals. In contrast, Bayes factors for three of the four correlations favored the data under alternative hypotheses over the null, with two suggesting moderate evidence and one indicating anecdotal evidence. The strongest of these Bayes factors, for the correlation between empty thresholds and slopes, is in agreement with the pattern of significance. Taken together, these findings are again inconsistent with a strong pacemaker hypothesis. However, a proponent might suggest that the pacemaker rate does determine performance on each task, but that participants’ thresholds and slopes for *empty* intervals are more greatly informed by pacemaker rate (for which the tasks correlate) than those for filled intervals (for which the tasks do not).

To test these inconsistencies from another angle, we again examined ordinal intra-individual differences in order to evaluate the implicit assumption that most participants would achieve their highest pacemaker rate for the same the stimulus type in each task. This proved true in this instance, with 79% of participants achieving their steepest slope and lowest threshold in the same stimulus type (all for filled intervals). This shows not only that the filled–empty effect is more pervasive than the modality differences in the two tasks we tested, but also lends some credibility to the idea that the task-independent pacemaker rate may in fact determine performance in both tasks. However, it is again worth nothing the differential effects of stimulus type on thresholds and slopes: Empty intervals led to slopes 30% shallower, and to thresholds 99% higher, than did filled intervals, on average. This was reflected in a higher effect size for filled–empty differences in thresholds (*d* = 1.19) than in slopes (*d* = 0.85). Therefore, there are still marked differences in effect sizes between the two tasks, illustrating probable differences in the decisions and processes involved.

## General discussion

In the present work we posited three research aims for testing the pacemaker explanation of modality differences and the filled-duration illusion in temporal difference thresholds and verbal estimation slopes. This explanation proposes that thresholds and estimation slopes are determined by a central pacemaker that runs at different rates for different modalities and stimulus types. For Research Aim 1, we closely replicated the threshold and estimation tasks of Jones et al. (2009). For Research Aim 2, we investigated the pervasiveness of modality differences and the filled-duration illusion by examining *inter*-individual differences. Finally, for Research Aim 3, we explored *intra*-individual differences by attempting to correlate thresholds and estimation slopes, and questioning whether the condition relating to participants’ highest pacemaker rates (and intermediate and lowest) was consistent across tasks. We will discuss the main findings from each experiment in order, and relate these to the relevant research aims.

Our replication of Jones et al.’ (2009) threshold task (our Exp. 1A) for Research Aim 1 showed that the thresholds for visual intervals were significantly higher than those for tactile and auditory intervals, whereas tactile thresholds were in turn significantly higher than auditory thresholds. This is consistent with the idea of a pacemaker that runs faster for auditory than for tactile intervals, and for tactile than for visual intervals. The investigation of inter-individual differences for Research Aim 2 showed that a lower auditory threshold, followed by an intermediate tactile threshold and a higher visual threshold, was the most common pattern (50%). Furthermore, 78% of participants exhibited the classic effect of lower thresholds for auditory than for visual intervals. This suggests that the pacemaker may indeed run faster for auditory than for visual intervals for most people, but even for this classic effect, the opposite may be true for around a fifth of people. Although we acknowledge the potential role of measurement error in obscuring participants’ “true” pacemaker rates, this approximation of participants’ exhibiting higher auditory than visual thresholds (the opposite of the classic effect) is nevertheless striking.

Experiment 1B continued our replication for Research Aim 1, and it showed that slopes were significantly steeper for auditory than for visual intervals, with no significant difference between auditory and tactile slopes. This is again consistent with the idea of a faster pacemaker for auditory than for visual intervals, though in this task there were no suggestions of a faster pacemaker for auditory than for tactile intervals. Exploration of inter-individual differences for Research Aim 2 showed that the most common pattern of slopes (37%) was a steeper tactile slope, followed by an intermediate auditory slope and a shallower visual slope. The most common pattern that had been found in thresholds, and indeed the pattern that presented in slopes at mean level (a steeper auditory slope, followed by an intermediate tactile slope, and a shallower visual slope), was the second most common, found in 31% of participants. Focusing on the classic auditory–visual difference, 73% of participant exhibited a steeper slope for auditory than for visual intervals, a similar percentage to that found in thresholds (78%).

Despite the similar patterns of results in Experiments 1A and 1B, no correlations were found for Research Aim 3A between thresholds and slopes (or between thresholds and predicted estimates of 700 ms), though theoretically they were determined by the same mechanism. This is therefore inconsistent with the pacemaker explanation. To consider the assumption from another angle, we questioned in Research Aim 3B whether the modality in which a participant performed best would be the same in both tasks (and also investigated intermediate and worst modalities). However, it was more common for participants to achieve their best performance (putatively the highest pacemaker rate) in different modalities in the two tasks. The same was true for intermediate performance. This contradicts an implicit assumption of the pacemaker explanation. On the other hand, most participants achieved their worst performance (putatively the lowest pacemaker rate) in the same modality in each task. Overall, however, the results were not complementary to the simple suggestion that a common pacemaker mechanism strongly determines both estimation slopes and thresholds.

To elucidate these findings, identical experiments were conducted to fulfill the same research aims in the context of the filled-duration illusion, which has a notably greater effect size than modality differences. In Experiment 2A, thresholds for empty intervals were found to be significantly higher than those for filled intervals (Research Aim 1), and 93% of participants appeared to exhibit this pattern, though of course an effect of measurement error might be possible (Research Aim 2).

In Experiment 2B, estimation slopes for filled auditory intervals were found to be significantly steeper than those for empty auditory intervals, with an effect size larger than the auditory–visual difference found in Experiment 1B. Exploration of inter-individual differences for Research Aim 2 found that 83% of participants appeared to exhibit this pattern, whereas the remaining participants were found to have steeper slopes for empty than for filled intervals. Our inter-individual difference findings cannot be directly compared to those of Hasuo et al. (2014), since those authors did not provide the number of participants whose estimation slopes were greater for filled than for empty intervals, or whose slopes were greater for empty than for filled intervals. Instead, Hasuo et al. (2014) found that one cluster (66%) of participants exhibited a small but significant underestimation of empty as compared to filled intervals, whereas the second cluster (34%) exhibited a clearer and much larger underestimation effect. We found that 83% of participants exhibited steeper slopes for filled than for empty intervals.

Bringing Experiments 2A and 2B together for Research Aim 3A, a correlation was found between thresholds and slopes for *empty* but not for filled intervals, though there was only a 4% difference in the amounts of variance explained in these two comparisons. We therefore feel that this small difference in the correlations for filled and empty intervals is insufficient to make any strong claims about the pacemaker, one way or the other. In terms of ordinal intra-individual differences, we found that 79% of participants achieved their steepest slope and lowest threshold for the same stimulus type (filled intervals).

If one would like to interpret the differential significance of the correlations for filled and empty intervals as theoretically important for the pacemaker, one would agree with the following interpretation: It could be that the pacemaker rate determines thresholds and slopes, but its contribution is much clearer for judgments of empty than of filled intervals. Perhaps mechanisms in addition to the pacemaker are used to inform time judgments in some tasks for *filled* intervals, as compared to when judging empty intervals. There might be some task-specific strategies for filled intervals that are only possible with a continuous sensory signal. For example, in verbal estimation, rather than judging each interval in its own right, participants might relate each duration to the previous one or to an internal standard that they have built up throughout the experiment. However, this additional working memory process might not be necessary or appropriate for the “which was longer” judgment of temporal difference thresholds, for either filled or empty intervals. When timing *empty* intervals, the task-specific strategy in verbal estimation might not be possible, or as easy, due to the lack of a continuous signal. Therefore, this might bring the estimation task in line with the threshold task in terms of its higher dependence on the pacemaker. This could explain the correlation between thresholds and slopes for empty but not for filled intervals.

Alternatively, if one were skeptical, either of the pacemaker or of the small difference in variance explained by the correlations for filled and empty intervals, one might favor this second interpretation: It could be that no correlations were found for the filled modalities in Experiment 1, whereas a correlation *was* found for empty intervals in Experiment 2, due to the greater variability present in the latter dataset. The standard deviation of empty slope values (0.25) was greater than those found in the other three conditions. Furthermore, whereas the standard deviation of empty threshold values (104.04) was not greater than that for visual thresholds (125.43), it might be the case that these two high standard deviations in tandem allowed for a significant correlation that might not have been found with less variable data (Goodwin & Leech, 2006).

It must be noted that although we have framed our investigations as a test of the pacemaker explanation, our work on inter- and intra-individual differences can be seen to test any hypothesis that seeks to explain the relationship between thresholds and slopes. It is possible that a nonpacemaker model could explain the slope effects observed in interval timing, but such an explanation has not yet been presented, to our knowledge.

Such an interpretation, complementary to the pacemaker, could be tested through a confirmatory factor analysis, in a manner similar to the investigation by Stauffer, Haldemann, Troche, and Rammsayer (2012). Indeed, our work could be repeated to compare the effects of filled and empty modality differences in a single experiment, to reduce variability and the effect of sampling error. In addition, further investigations of the impacts of the pacemaker on thresholds and slopes could use intermittent intervals. These intervals might act as a halfway house between filled and empty intervals, so that the contribution of the pacemaker might be clearer than when using continuous filled intervals. It might be that enough consistent sensory information would not be present for alternative strategies to work on, so correlations could be found between thresholds and slopes for intermittent intervals. Finally, future work might wish to create a computational model of the temporal difference threshold task according to scalar timing theory, in order to allow for investigations of whether a mathematical increase in pacemaker rate does in fact produce a reduction in thresholds, as was suggested by Jones et al. (2009). Ulrich, Nitschke, and Rammsayer (2006) extended a general pacemaker–accumulator model created by Rammsayer and Ulrich (2001) in order to model the effect of differences in pacemaker rate on thresholds. However, their pacemaker was specified in terms of a (distribution-free) formula from renewal theory, rather than by using the Poisson pacemaker with trial-to-trial variability from scalar timing theory.

The results of our explorations of inter-individual differences add weight to recent developments in the literature. As was highlighted by Matthews and Meck (2014) in their review of the current state of the timing field, some of the classic effects or illusions in time perception may not be as robust as they appear. We have already mentioned the work by Hasuo and colleagues (Hasuo et al., 2014; Hasuo et al., 2011), who found that the size of the filled-duration illusion varied greatly in magnitude between participants, with some participants even showing a reversal of the effect. Our findings for filled and empty intervals are in agreement with this. Additionally, we have now shown that the same is true of the totemic effect of modality, for both thresholds and estimates. To our knowledge, this is the first time this has been considered.

In summary, our research to test the pacemaker explanation can be summarized according to our three research aims. First, we extended the work of Jones et al. (2009) by replicating their threshold and estimation task, with one group of participants for modality differences, and with another group for filled-duration illusions. Second, though modality differences and the filled-duration illusion are robust and sizeable effects, we found that up to a quarter of people may present opposite patterns to those classically found. Third, and finally, correlations conducted between estimation slopes and thresholds did not offer consistent evidence in line with the suggestion that the pacemaker strongly determines performance on both tasks. In addition, it appeared more common for participants to perform best in different modalities in each task, though this did not appear true of ordinal intra-individual differences in the filled-duration illusion. Overall, the pacemaker rate explanation appears to be inconsistent with several findings in the present research.

## Electronic supplementary material

ESM 1 (DOCX 107 kb)

## Footnotes

1 We refer readers to Grondin (2010) for an overview of alternative models of time perception.

2 “Verbal” estimation is a misnomer, which comes from the assignment of verbal labels (e.g., “seconds”) to the duration of intervals, rather than from a request for verbal (i.e., dictated) responses.

3 Procedures for establishing temporal difference thresholds vary greatly, but this finding is consistent across similar incarnations of the task (e.g., same standard duration, adaptive vs. nonadaptive procedure).

4 Jones et al. (2009) had recruited 28 participants for their threshold task and 22 participants for their estimation task. We recruited 52 participants to complete both of these tasks, in order to have increased confidence and statistical power.

5 The single number rating (SNR) is the European equivalent of the noise reduction rating (NRR) in the United States.

6 Due to a programming error, the durations of our intended standard and comparisons were inverted. Jones et al.’s (2009) standard duration was 1,000 ms with an initial comparison duration of 700 ms. This will be taken into account when comparing the results of the two studies.

7 The raw and aggregate data are available online in an Open Science Framework repository: https://osf.io/gre73/ (10.17605/OSF.IO/GRE73).

8 Though set a priori, this criterion was proved functional by preserving apparently natural thresholds (including those 500–600 ms) in Experiments 1A and 2A, and highlighting apparently erroneous thresholds (upward of 1,000 ms) in Experiment 2A.

9 See the supplementary material for nonparametric tests confirming these results, given the significant deviations of the residuals from normality (Shapiro–Wilks *p* < .001 for auditory, *p* = *.*02 for tactile, and *p* = .019 for visual thresholds).

10 As was suggested by a reviewer, Holm’s (1979) sequentially rejective Bonferroni test is at least equal in power to the standard Bonferroni procedure, and oftentimes is more powerful. First, each *p* value of a set of comparisons is ranked from lowest to highest. A unique alpha criterion is then calculated for each comparison as [*a*/(*n* – rank – 1)], where *a* is the original alpha level and *n* is the number of comparisons. The *p* values of the comparisons are then checked against the new alpha criteria in order of rank, but once one *p* value is found to be greater in value than the criterion, this and all following comparisons are considered nonsignificant.

11 Bayes factors are reported for each *t* test, calculated using JASP (JASP Team, 2017). The notation used is BF_10_ (two-tailed), BF_–0_ (one-tailed hypothesis that the first-named is lower), or BF_+0_ (one-tailed hypothesis that the first-named is higher). These three options report the ratio of the probability of the data under the alternative hypothesis to its probability under the null hypothesis. For Bayes factors reporting the probability of the data in favor of the null hypothesis, these notations are inverted (i.e., BF_01_, BF_0–_, and BF_0+_, respectively) and represent the number of times the null hypothesis is more likely than the alternative hypothesis. The interpretation of Bayes factors is taken from Lee and Wagenmakers (2013).

12 Interestingly, both participants achieved the same value for their auditory and visual thresholds (44 ms for P11 and 114 ms for P51, with tactile thresholds of 64 and 42 ms, respectively). Therefore, P11’s threshold pattern was [A = V < T], whereas P51’s threshold pattern was [T < A = V].

13 These durations were originally selected in order to avoid artificial accuracy. Participants’ estimates are typically quantified and usually end in “50” or “00,” in addition to a high frequency of 250, 500, 750, or 1,000 (Wearden, 2015). Therefore, presenting intervals in these durations might result in participants spuriously estimating the duration correctly.

14 P47’s estimation function for visual intervals was not significant (*p* = .503), with a slope of 0.05 and an intercept of 134 ms. This participant’s auditory function had a slope of 0.58 and an intercept of 19 ms, whereas their tactile function had a slope of 0.52 and an intercept of – 56 ms.

15 See the supplementary material for nonparametric tests confirming these results, given significant deviations of the residuals from normality (Shapiro–Wilks *p* < .001 for each condition).

16 P47 was excluded from this analysis because their visual slope was not significant. Their slope pattern was [A > T > *v*].

17 It would be unreasonable to expect to find this congruency effect for all participants, because even if the effect *were* true for all participants, it would likely be masked by some amount of measurement error.

18 The interested reader can find correlations between the thresholds and coefficients of variation of the estimates for both experiments in the supplementary material.

19 These participants’ threshold patterns were [A = V < T] for P11, [A < T < V] for P47, and [T < A = V] for P51, whereas respective slope patterns were [V > A > T], [A > T > *v*], and [A > T > V].

20 We recruited 32 participants, in contrast to the 52 in Experiment 1, due to the larger effect size for filled versus empty intervals. Our estimation procedure for filled versus empty intervals was almost exactly that of Wearden et al. (2007), who had recruited 15 participants. Although, in error, no ages were collected for these participants, all participants in this sample were from the undergraduate population.

21 Both P56’s and P58’s thresholds for empty intervals were much greater than 600 ms: 1,136 and 1,212 ms, respectively. Their respective filled thresholds were 174 and 78 ms.

22 See the supplementary material for nonparametric tests confirming these results, given significant deviations of the residuals from normality (Shapiro–Wilks *p* < .001 for filled thresholds).

23 P58’s estimation function for filled intervals was not significant (*p* = .858), with a slope of 0.03 and an intercept of 675 ms. Similarly, their estimation function for empty intervals was not significant (*p* = .493), with a slope of – 0.07 and an intercept of 763 ms. P64’s estimation function for empty intervals was also not significant (*p* = .335), with a slope of 0.07 and an intercept of 107 ms, whereas their function for filled intervals was significant, with a slope of 0.41 and an intercept of – 5 ms.

24 See the supplementary material for a figure presenting filled–empty estimation difference values, along with those for modality differences.

25 See the supplementary material for nonparametric tests confirming this result, given the significant deviations of the residuals from normality (Shapiro–Wilks *p* < .001 for filled and *p* = *.*02 for empty intercepts).

26 P58’s slope pattern was [*f* > *e*], though neither was significant. Similarly, P64’s slope pattern was [F > *e*], though their empty slope was not significant.

27 These participants’ threshold patterns were [F < *e*] for P56, [F < *e*] for P58, and [F < E] for P64, whereas the respective slope patterns were [F > E], [*f* > *e*], and [F > *e*].
