# Supplementary material for: Modality differences in timing and the filled-duration illusion: Testing the pacemaker rate explanation
Source: Atten Percept Psychophys. 2018 Dec 19;81(3):823–45. doi: 10.3758/s13414-018-1630-8 (PMC6407723; doi:10.3758/s13414-018-1630-8)
Supplement: Supplementary file 1 — (DOCX 107 kb) [file 13414_2018_1630_MOESM1_ESM.docx]

**Supplementary Material**

Non-Parametric Analyses of Auditory, Tactile and Visual Thresholds

A Friedman test found a significant difference between thresholds for the different modalities, *χ*^2^_(2)_ = 28.12, *p* < .001. Post hoc Wilcoxon Signed Ranks tests (Bonferroni-corrected, *α* = .017) confirmed that thresholds for visual intervals were significantly higher than auditory (*Z* = 4.95, *p* < .001) and tactile intervals (Z = 4.62, *p* < .001). However, thresholds for auditory and tactile intervals did not significantly differ (Z = 2.25, *p* = .024).

Non-Parametric Analyses of Auditory, Tactile and Visual Intercepts

A Friedman test found a significant difference between intercepts for the different modalities, *χ*^2^_(2)_ = 9.27, *p* = .010. Post hoc Wilcoxon Signed Ranks tests (Bonferroni-corrected, *α* = .017) confirmed that the intercepts for tactile durations were significantly lower than intercepts for visual (*Z* = 3.43, *p* = .001) and auditory durations (*Z* = 2.42, *p* = .015). Intercepts for auditory and visual durations did not significantly differ (*Z* = 1.24, *p* = .216).

Correlations for Auditory, Tactile and Visual Thresholds and Coefficients of Variation of Estimates

Following a reviewer’s suggestion, we have calculated correlations between thresholds and the coefficients of variation (CVs) of estimates. The rationale here is that both thresholds and CVs of estimates can be thought of as indexes of the variability of temporal representations. Though we agree with this in principle, we refrain from including these in the main text due Wearden’s (2015) finding that that measured CVs of estimates are not a reliable indicator of the CV of the internal representation of duration, based on his model of verbal estimation. In addition, our aim was to test the assertion that both thresholds and estimation *slopes* are indexes of pacemaker speed. Therefore, a search for additional correlating variables was decided to be outside the remit of the current paper. Nevertheless, we include them here as an additional piece of the puzzle.

We have calculated CVs as the standard deviation of the five estimates at a given stimulus duration within a given modality, divided by the mean of those estimates. We then took the mean of these duration-specific CVs in order to calculate one CV value for each modality. See Table 1 for these results.

**Table 1** Pearson correlations between Temporal Difference Thresholds and Coefficients of Variation of Estimates in Experiment 1.

| Threshold |  | Coefficients of Variation of Estimates | | | |
| --- | --- | --- | --- | --- | --- |
|  |  | *n* | *r* | *p* | *BF_0-_* |
| Auditory |  | 52 | -.079 | .578 | 3.51 |
| Tactile |  | 52 | -.010 | .944 | 5.47 |
| Visual |  | 51 | -.428 | .002 | 0.02 |

*Note:* Bayes factors express the amount of evidence in favour of the data given the null hypotheses.

Non-Parametric Correlations for Auditory, Tactile and Visual Thresholds and both Estimation Slopes and 700ms Estimates

See Table 2 for non-parametric correlations between temporal difference thresholds, estimation slopes and 700 ms estimates from Experiment 1.

**Table 2** Spearman correlations between temporal difference thresholds and both estimation slopes and 700 ms estimates in Experiment 1.

| Threshold |  | Estimation Slope | | |  | 700 ms Estimates | | |  |
| --- | --- | --- | --- | --- | --- | --- | --- | --- | --- |
|  |  | *n* | *r* | *p* |  | *n* | *r* | *p* | |
| Auditory |  | 52 | -.076 | .590 |  | 52 | -.086 | .542 | |
| Tactile |  | 52 | -.204 | .147 |  | 52 | -.056 | .692 | |
| Visual |  | 51 | -.229 | .106 |  | 51 | -.071 | .619 | |

*Note:* *α* = .025 for each column.

Non-Parametric Analyses of Filled and Empty Thresholds

A Wilcoxon Signed Rank test confirmed that the thresholds for filled durations were significantly lower than those for empty durations (*Z* = 4.54, *p* < .001).

Non-Parametric Analyses of Filled and Empty Intercepts

A Wilcoxon Signed Rank test confirmed that the intercepts for filled and empty did not significantly differ (*Z* = 0.36, *p* = .719).

Correlations for Filled and Empty Thresholds and Coefficients of Variation of Estimates

See Table 3 for correlations between temporal difference thresholds and coeffecients of variation of estimates from Experiment 2.

**Table 3** Pearson correlations between Temporal Difference Thresholds and Coefficients of Variation of Estimates in Experiment 2.

| Threshold |  | Coefficients of Variation of Estimates | | | |
| --- | --- | --- | --- | --- | --- |
|  |  | *n* | *r* | *p* | *BF_0-_* |
| Filled |  | 31 | -.550 | .001 | 0.02 |
| Empty |  | 30 | -.382 | .037 | 0.28 |

*Note:* Bayes factors express the amount of evidence in favour of the data given the null hypotheses.

Non-Parametric Correlations for Filled and Empty Thresholds and both Estimation Slopes and 700ms Estimates

See Table 4 for non-parametric correlations between temporal difference thresholds, estimation slopes and 700 ms estimates from Experiment 2.

**Table 4** Spearman correlations between temporal difference thresholds and both estimation slopes and 700 ms estimates in Experiment 2.

| Threshold |  | Estimation Slope | | |  | 700 ms Estimates | | |
| --- | --- | --- | --- | --- | --- | --- | --- | --- |
|  |  | *n* | *r* | *p* |  | *n* | *r* | *p* |
| Filled |  | 31 | -.352 | .052 |  | 31 | -.383 | .034 |
| Empty |  | 29 | -.443 | .016 |  | 29 | -.188 | .328 |

*Note:* α = .025 for each column.

Difference Values for Estimates in Experiments 1 and 2

Upon the suggestion of a reviewer, we present the difference values for each condition in the verbal estimation task (see Figure 1). We first removed participants who had been excluded previously (e.g. P47 from the visual estimates), averaged all remaining participants’ estimates for each stimulus duration, then subtracted the relevant conditions at this mean level. True to the reviewer’s suspicion, it is not always the case that difference values increase as a function of stimulus duration. This is especially notable in the Filled – Empty difference condition, where the difference increases quite drastically for stimulus durations of up to 767 ms, then decrease thereafter. This suggests that there is a slope difference up to this value, after which linear functions are then parallel (as can be seen in Figure 10 in the paper).


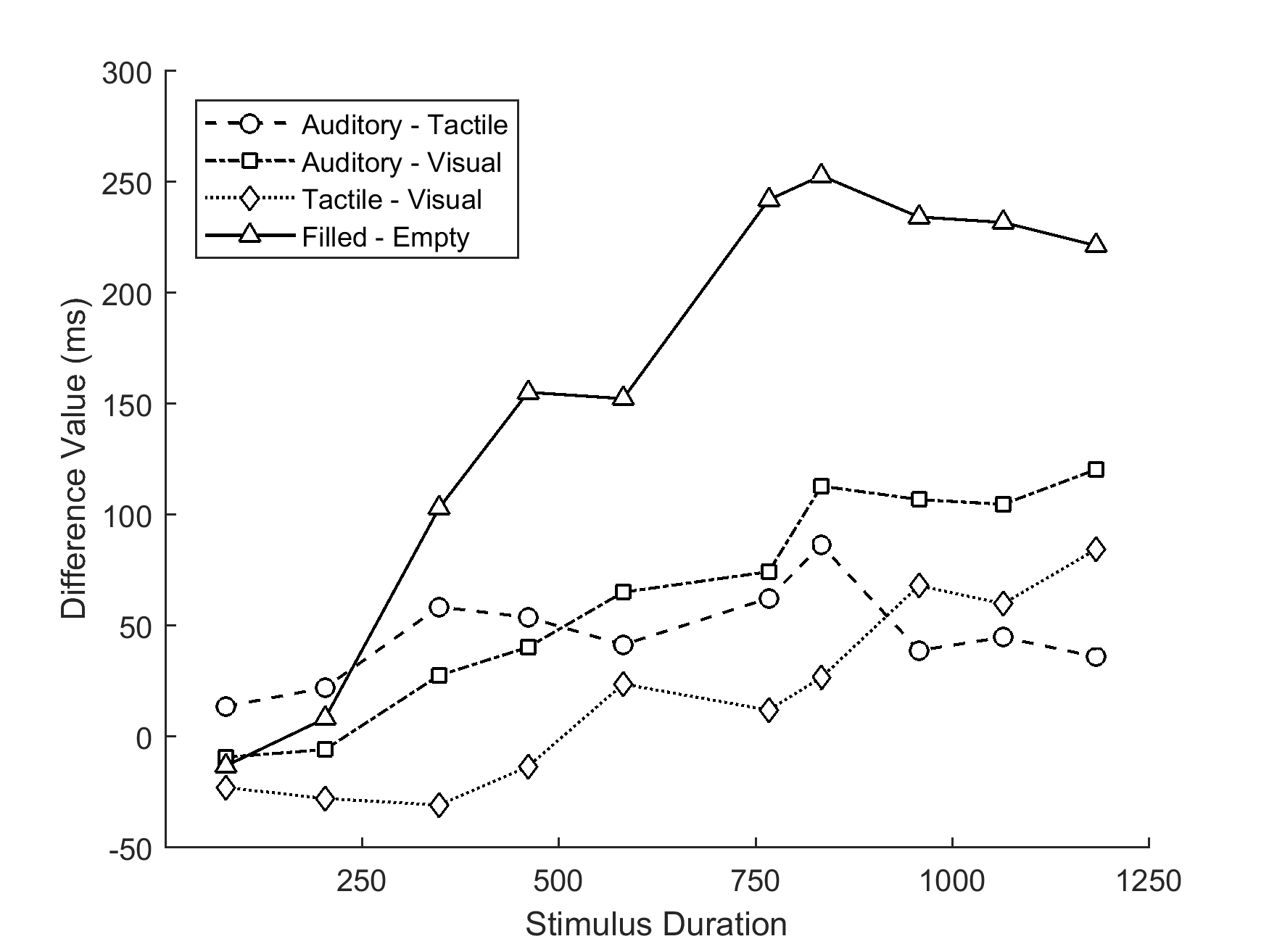


**Fig. 1** Difference values of estimates for each condition in Experiments 1 and 2.
